# RNA-seq of Rice Yellow Stem Borer *Scirpophaga incertulas* Reveals Molecular Insights During Four Larval Developmental Stages

**DOI:** 10.1534/g3.117.043737

**Published:** 2017-07-17

**Authors:** Pichili Renuka, Maganti S. Madhav, Ayyagari Phani Padmakumari, Kalyani M. Barbadikar, Satendra K. Mangrauthia, Kola Vijaya Sudhakara Rao, Soma S. Marla, Vemuri Ravindra Babu

**Affiliations:** * Department of Biotechnology, ICAR-Indian Institute of Rice Research, Hyderabad, Telangana 500030, India; † Department of Entomology, ICAR-Indian Institute of Rice Research, Hyderabad, Telangana 500030, India; ‡ Division of Genomic Resources, ICAR-National Bureau of Plant Genomic Resources, New Delhi, India; § Department of Plant Breeding, ICAR-Indian Institute of Rice Research, Hyderabad, Telangana 500030, India

**Keywords:** insect, *Scirpophaga incertulas*, *de novo* transcriptome, RNAi, growth and development, detoxification mechanism

## Abstract

The yellow stem borer (YSB), *Scirpophaga incertulas*, is a prominent pest in rice cultivation causing serious yield losses. The larval stage is an important stage in YSB, responsible for maximum infestation. However, limited knowledge exists on the biology and mechanisms underlying the growth and differentiation of YSB. To understand and identify the genes involved in YSB development and infestation, so as to design pest control strategies, we performed *de novo* transcriptome analysis at the first, third, fifth, and seventh larval developmental stages employing Illumina Hi-seq. High-quality reads (HQR) of ∼229 Mb were assembled into 24,775 transcripts with an average size of 1485 bp. Genes associated with various metabolic processes, *i.e.*, detoxification mechanism [CYP450, GSTs, and carboxylesterases (CarEs)], RNA interference (RNAi) machinery (*Dcr-1*, *Dcr-2*, *Ago-1*, *Ago-2*, *Sid-1*, *Sid-2*, *Sid-3*, and *Sid-1*-related gene), chemoreception (CSPs, GRs, OBPs, and ORs), and regulators [transcription factors (TFs) and hormones] were differentially regulated during the developmental stages. Identification of stage-specific transcripts made it possible to determine the essential processes of larval development. Comparative transcriptome analysis revealed that YSB has not evolved much with respect to the detoxification mechanism, but showed the presence of distinct RNAi machinery. The presence of strong specific visual recognition coupled with chemosensory mechanisms supports the monophagous nature of YSB. Designed expressed sequenced tags-simple-sequence repeats (EST-SSRs) will facilitate accurate estimation of the genetic diversity of YSB. This is the first report on characterization of the YSB transcriptome and the identification of genes involved in key processes, which will help researchers and industry to devise novel pest control strategies. This study also opens up a new avenue to develop next-generation resistant rice using RNAi or genome editing approaches.

Rice Yellow Stem Borer (YSB), *Scirpophaga incertulas* (Walker) (Lepidoptera: Crambidae) is the most destructive insect pest of rice found in diverse ecosystems across the world. It has been reported to be the dominant pest in Asia (Listinger 1979), South East Asia (Banerjee and Pramanik 1967), and India in particular (Chelliah *et al.* 1989). It is predominantly a monophagous pest and there are no reports that it can successfully complete its life cycle on any other plant outside the species *Oryza* (Pathak and Khan 1994). YSB attacks the rice plant from seedling to maturity stage, resulting in the formation of dead hearts and white ears. Yield losses due to YSB damage are significant and 1% each of dead hearts and white ears leads to yield losses of 2.5 and 4.0%, respectively (Muralidharan and Pasalu 2006). The application of chemical insecticides at the seedling and reproductive stages of rice is effective and therefore widely adopted practice in the management of YSB. However, continuous use of pesticides causes health and environmental hazards (Rath 2001). Hence, there is a need to search for alternative strategies to combat this deadly pest. To control YSB, it is essential to understand the biochemical and molecular mechanisms associated with its life cycle. The process of exploring pest genomic information for the design of control strategies has been progressive, but limited to only some pests. Genome-wide expression analysis of genes during the different developmental stages of the insect will provide molecular insights into its life cycle. This will eventually aid the identification of key insect transcripts involved in infestation and growth that can be targeted for the development of resistance against this pest (Kola *et al.* 2015).

With the advent of next-generation sequencing platforms, obtaining biological perspectives of cellular processes has become a reality. Transcriptome analysis through RNA-seq can be utilized efficiently to identify the temporal and unique gene expression patterns in an organism (Ozsolak and Milos 2011). Specifically, it provides novel opportunities for expression studies in organisms lacking genome or transcriptome sequence information (Haas and Zody 2010; Wolf 2013). RNA-seq has been used extensively in insect pests to reveal biological phenomena (Ou *et al.* 2014), gene expression profiles, and gene discovery (Vogel *et al.* 2014). It has also been used to identify RNAi targets to develop pest control strategies (Wang *et al.* 2011). The developmental transcriptome studies in insect pests have provided better understanding of the transition phases of the insect life cycle. RNA-seq data also offers a great opportunity to develop genomic resources, namely the EST-SSRs leveraged by the function of the transcripts. Such functional markers act as a repository of genomic resources for the insect species (Stolle *et al.* 2013). The larval stage is the important developmental stage in YSB, being responsible for economic damage. In the present study, with the aim of understanding and identifying the temporal and unique gene expression patterns during the larval stages in YSB, we performed RNA-seq at the first, third, fifth, and seventh instars. A total of 236 million reads were generated and *de novo* assembled into 24,775 transcripts of YSB. The differentially expressed transcripts were analyzed by comparing gene expression profiles and further validated using qRT-PCR. We also performed comparative transcriptome analysis with related insect pest species for better understanding of behavioral and evolutionary aspects of YSB. Functional EST-SSR markers were also developed for YSB, which will serve as a rich genetic resource for diversity studies. These findings will facilitate future molecular studies aim at understanding the biology of YSB and related insects. Such YSB-specific functionally important transcripts may be used as target genes for RNAi to develop YSB resistance in rice.

## Materials and Methods

### Insect rearing and sample collection

The YSB adults were collected from Indian Council of Agricultural Research-Indian Institute of Rice Research paddy fields in the wet season 2013. The insects were released on rice plants (TN1 variety), and maintained under controlled conditions at 25 ± 2° temperature and 80% relative humidity for oviposition. Egg masses laid on the leaf blades were collected and placed in glass vials for hatching. Neonate larvae were reared on cut stems of rice plants (TN1 variety). Different larval instars, namely the first, third, fifth, and seventh (L_1_, L_3_, L_5_, and L_7_, respectively) were identified on the basis of mandibular width and visual observation of exuvae (Padmakumari *et al.* 2013). Each larval instar was collected 2–4 hr after moulting in three replicates and stored in RNA later (Invitrogen) at −80° for total RNA isolation.

### RNA sequencing

**Figure 1:**
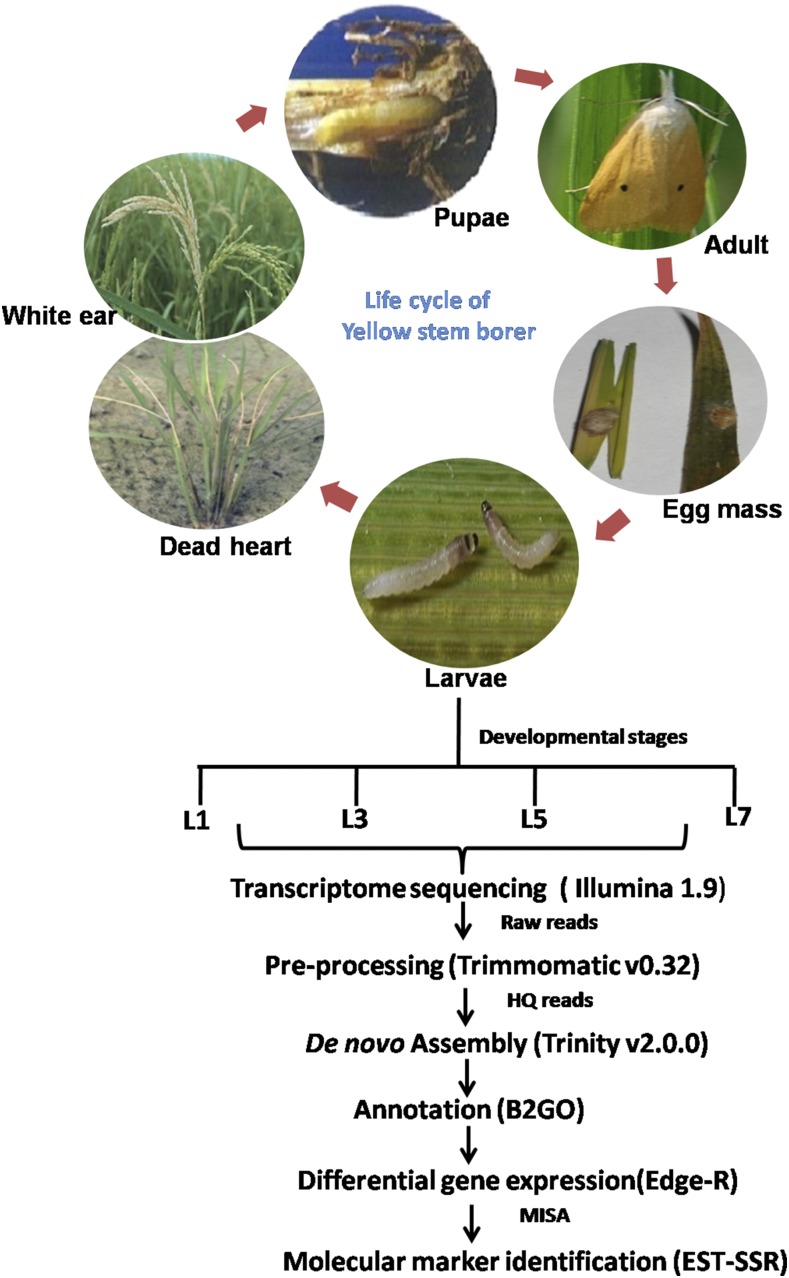
Schematic representation of *S. incertulas* (yellow stem borer) transcriptome sequencing. EST-SSR, expressed sequenced tags-simple-sequence repeats; HQ, high-quality; MISA, MIcroSAtellite identification tool.

Total RNA was isolated using the SV total RNA isolation system (Promega) as per the manufacturer’s protocol. The RNA was pooled from three replicates of each instar. The yield and purity of RNA were assessed by measuring the absorbance at 260 and 280 nm, and quality was checked on a formaldehyde gel using MOPS buffer. The RNA integrity number (RIN) was checked by running the RNA Nano chip on a Bioanalyzer (Agilent Technologies) and samples with a RIN value > 7.5 were processed further. As required for Illumina sequencing (San Diego, CA), mRNA was isolated from 5 μg of total RNA and paired-end (2 × 100 bp) cDNA sequencing libraries were prepared using an Illumina TruSeq RNA Library Preparation Kit as per the manufacturer’s protocol (Illumina) and sequenced on a HiSequation 2000 sequencing system at Nucleome Informatics Pvt.Ltd., Hyderabad, India. The schematic representation of YSB *de novo* transcriptome analysis is given in Figure 1.

### Preprocessing of raw data and read mapping

The YSB raw reads were filtered to obtain HQR by removing low-quality reads and adaptors using Trimmomatic v0.32 with default parameters. The resulting HQR from all the four samples were assembled using Trinity v2.0.0 with default parameters (http://trinityrnaseq.sourceforge.net/, Haas *et al.* 2013). The redundancy of the assembly was reduced through processing by Cd-hit (Li and Godzik 2006), TGICL (Pertea *et al.* 2003), and Evigene (http://arthropods.eugenes.org/EvidentialGene/about/EvidentialGene_trassembly_pipe.html). The *de novo* assembled transcripts were named as YSB_LS as prefixed by the number emanated from Trinity assembler.

### Functional annotation of transcripts

The *de novo* assembled transcripts were subjected to BLASTx against the protein nonredundant database, Swiss-Prot, and the Kyoto Encyclopedia of Genes and Genomes (KEGG) database with an *E*-value cut-off of 10^−5^. Gene Ontology (GO) functional annotation of transcripts was performed using the Blast2GO server (Conesa *et al.* 2005). Pathway mapping was executed for transcripts using the KEGG (www.genome.jp/kegg) database with an *E*-value cut-off of 10^−5^.

### Differential expression of transcripts

The differentially expressed transcripts between the four larval stages were analyzed using EdgeR, available as a plug-in in Trinity based on the FPKM (Fragments Per Kilobase of transcript per Million mapped reads) value (Robinson *et al.* ‎2010). Transcripts expressed at very low levels (read counts < 10 across all four libraries) were not considered and *P*-value ≤ 0.05 was used to identify differentially expressed transcripts. The significance of differences in gene expression was judged using a threshold FDR ≤ 0.001 and an absolute value of log_2_ ratio > 1. Heat maps showing differential expression of transcripts belonging to TF families, RNAi machinery, and chemoreception were generated by using MeV 4.0 (https://sourceforge.net/projects/mev-tm4/) software. Heat maps were prepared based on relative FPKM values using hierarchical clustering based on average Pearson’s distance following the complete linkage method (Saeed *et al.* 2003).

### Expression validation of transcripts by qRT-PCR

The differentially expressed transcripts selected based on their function were validated through qRT-PCR. An aliquot of 1 μg total RNA was reverse transcribed into single-stranded cDNA using the Prime Script RT reagent kit (TaKaRa, Japan). Primers for qRT-PCR were designed using online software Primer 3 (http://primer3.ut.ee/) and synthesized from IDT (Supplemental Material, Table S1). The qRT-PCR primers were checked for the presence of hairpin structures or dimer formation using the online Oligo Analyzer 3.1 tool (http://eu.idtdna.com/calc/analyzer; IDT). The reaction mixture for qRT-PCR comprised the SYBR premix Ex-Taq kit (TaKaRa, Japan) with 2 µl normalized cDNA template and 10 pg forward and reverse primers each. Reactions were performed in PCR LC-96-well plates (Roche LightCycler 96; Roche). The YSB β-actin gene reported in our previous study was used as an internal control (Kola *et al.* 2016). The relative gene expression was analyzed by the ΔΔCT method and fold change was calculated by 2^−ΔΔCT^ (Schmittgen and Livak 2008).

### EST-SSR mining

The YSB *de novo* assembled transcripts were subjected to SSR motif identification using the MIcroSAtellite identification tool (MISA; http://pgrc.ipkgatersleben.de/misa/) and henceforth called YSB EST-SSRs. The following parameters were used for the identification of dinucleotide repeats (2/6), trinucleotide repeats (3/5), tetranucleotide repeats (4/5), pentanucleotide repeats (5/5), and hexa-nucleotide repeats (6/5). The SSR motifs were classified based on the type of repeat classes and the presence of repeat motifs. Three different sets of EST-SSR primers were designed using Batch primer 3, having a Tm of 55–60°, GC content of 40–50%, and amplicon size ranging between 100 and 280 bp (http://probes.pw.usda.gov/cgi-bin/batchprimer3/batchprimer3.cgi; You *et al.* 2008). The list of newly developed YSB EST-SSR primers is provided in Table S2.

### Comparative transcriptome analysis with other relevant pests

A comparative transcriptome analysis was carried out by comparing the protein sequences of the YSB transcriptome with related lepidopteran and hemipteran insect genomes. From the National Centre for Biotechnology Information (NCBI) ftp site (www.ncbi.nlm.nih.gov/ftp), protein sequences of related insect species from genomes of *Chilo suppressalis* (striped rice stem borer), *Cnaphalocrocis medinalis* (rice leaf folder), and *Nilaparvata lugens* [brown plant hopper (BPH)] (reported rice pests); *Spodoptera frugiperda* (fall armyworm) and *Helicoverpa armigera* (American boll worm) (two polyphagous lepidopteran pests); and *Bombyx mori* (silkworm) (a monophagous pest) were retrieved. Comparison of protein sequences was done using the BLASTx against the above-mentioned pest genomes with an *E*-value cut off of 10^−5^ and 80% similarity.

### Phylogenetic analysis

Phylogenetic analysis was carried out for the transcripts belonging to some of the important functional groups, namely insecticide detoxification, chemoreception, and RNAi machinery. For this analysis, transcripts of lepidopteran, hemipteran, and coleopteran sequences were retrieved from NCBI (February 10, 2016). The phylogenetic tree was constructed from the multiple alignments using MEGA 5.0 generated with 1000 bootstrap trials by the Neighbor-Joining method (Tamura *et al.* 2007).

### Data availability

The YSB raw reads and *de novo* assembled YSB transcripts are available in the Short Read Archive with the accession number SRX733621, and the Transcriptome Shotgun Assembly at the NCBI.

## Results and Discussion

### Sequencing and de novo assembly

Sequencing of four larval stages yielded a total of 236 million reads, ranging from 32 to 83 million per library. The raw reads were filtered to remove adaptors and sequencing artifacts prior to assembly. A total of 229 million (97.3%) cleaned reads (after preprocessing and quality checking) were obtained from raw reads and the corresponding statistics are presented in Table 1. Since, a reference genome is not available for YSB, *de novo* transcriptome assembly was done using a Trinity assembler. Assembly of HQR resulted into 24,775 transcripts with a total length of 36.77 Mb. The average length of YSB transcripts was 1485 bp with N50 of 2348 bp (Table 2).

**Table 1:** Summary of raw and high-quality reads of *S. incertulas* transcriptome data

Larval Stages	Raw Reads	High-Quality Reads
L_1_	32,799,252	32,762,167
L_3_	82,291,698	80,036,367
L_5_	38,018,874	37,977,644
L_7_	83,347,094	79,215,820
Total	236,456,918	229,991,998

L_1_, first instar larvae; L_3_, third instar larvae; L_5_, fifth instar larvae; L_7_, seventh instar larvae.

**Table 2:** Statistics of *de novo* assembled *S. incertulas* transcriptome

Description	Value
Number of transcripts	24,775
Total size of transcripts (bp)	36,779,612
Longest transcripts (bp)	39,712
Shortest transcripts (bp)	201
Number of transcripts > 1 Knt	12,404 (50.1%)
Number of transcripts > 10 Knt	49 (0.2%)
Mean transcript size	1,485
Median transcripts size	1,004
N50 (bp)	2,348
GC content (%)	44

Knt, kilo nucleotides.

The Illumina platform has been widely used for RNA-seq on account of its large data size, precision, and ease (Porreca *et al.* 2007). The huge amount of raw data generated in RNA-seq needs preprocessing and quality checking to remove sequence artifacts, low complexity reads, and adaptor contamination for better *de novo* assembly (Patel and Jain 2012). The preprocessing and assembly of data were carried out by Cd-hit, TGICL, and Evigene, which efficiently handles long reads or transcripts and gives nonredundant data as evidenced by earlier studies (Zhao *et al.* 2016; Zhang *et al.* 2014). Although *de novo* transcriptome assembly without a reference genome represents a computational challenge (Kulkarni *et al.* 2016), Trinity is an efficient tool for robust rebuilding of transcriptomes from large volumes of RNA-seq data, especially those of ecological and evolutionary importance (Grabherr *et al.* 2011). Trinity has been successfully employed in *de novo* assembly of other insect transcriptomes, namely *C. suppressalis* (Yin *et al.* 2014) and *Vespa mandarinia* (Patnaik *et al.* 2016).

**Figure 2:**
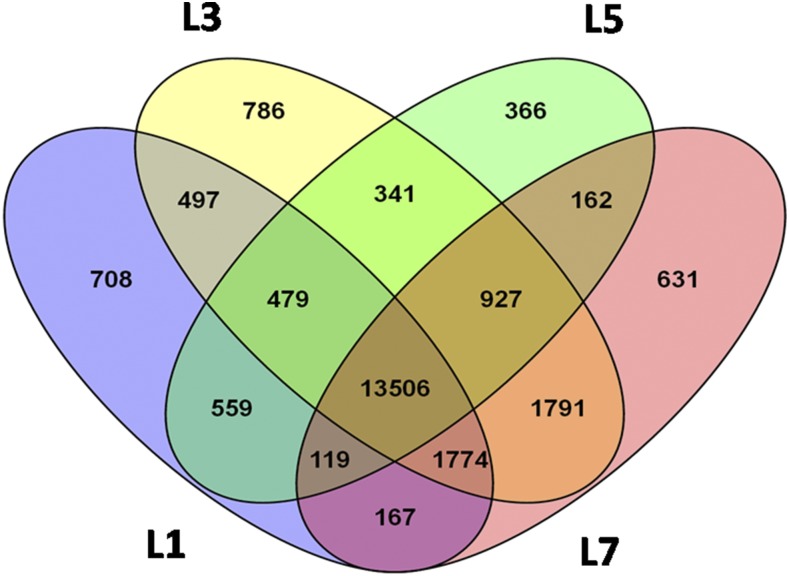
Venn diagram depicting the number of *S. incertulas* transcripts in each larval developmental stage. L_1_, first instar larvae; L_3_, third instar larvae; L_5_, fifth instar larvae; L_7_, seventh instar larvae.

Twenty-four thousand, seven hundred and seventy-five transcripts obtained in this study were compared with transcriptome data of other related rice insects, namely *C. medinalis* (44,941 unigenes) (Li *et al.* 2012) and *C. suppressalis* (37,040 contigs) (Ma *et al.* 2012). The transcripts emanating from *de novo* assemblies depend on distinct quality parameters and thus differ according to the parameters employed in each study (Schliesky *et al.* 2012). The N50 value of the YSB transcriptome was higher compared to previously reported insect transcriptome assemblies, which indicated the maximum utilization of reads. The 44% GC content of the YSB transcriptome corroborated earlier reports showing GC content between 40 and 48% (Camargo *et al.* 2015; Li *et al.* 2013). Out of the 24,775 assembled transcripts, 13,506 transcripts were commonly expressed in all developmental stages, while 708, 786, 366, and 631 transcripts were specific to L_1_, L_3_, L_5_, and L_7,_ respectively (Figure 2). From our study, the transcripts required for basal metabolic activities like cuticular formation, energy releasing mechanisms, and digestion were commonly expressed. It is evident that larval development involves a set of essential metabolic activities for its growth and development, while the stage-specific transcripts might be important for specific development at a particular larval stage (Ou *et al.* 2014).

### Functional annotation of YSB transcripts

The maximum number of YSB transcripts were annotated with Swiss-prot (30132), followed by Blast2GO (24775) and KEGG (1078). The results obtained from functional annotation of the total 24,775 transcripts with BLASTx against the NCBI nr database are presented in Table S3. Functional distribution of transcript data with Blast2GO is given in Figure S1. The maximum number of YSB transcripts matched with lepidopteran pests, namely silk worm (*B. mori*, 41.3%), monarch butterfly (*Danus plexippus*, 27.2%), Asian swallowtail (*Papilio xuthus*, 1.5%), American boll worm (*H. armigera*, 0.62%), pink bollworm (*Pectinophora gossypiella*, 0.53%), fall armyworm (*S. frugiperda*, 0.3%), and European corn borer (*Ostrinia nubilalis*, 0.24%). Some transcripts of YSB also matched, but to a lesser extent with other insect species such as red flour beetle (*Tribolium castaneum*, 4.2%), pea aphid (*Acyrthosiphon pisum*, 3.3%), Asian citrus psyllid (*Diaphorina citri*, 3.0%), *Microplitis demolitor* (1.4%), clonal raider ant (*Cerapachys biroi*, 1.2%), and jewel wasp (*Nasonia vitripennis* 0.81%). Interestingly, only 0.9% of YSB transcripts matched to the striped rice stem borer (*C. suppressalis*) and even less (0.24%) with BPH (*N. lugens*). However, the maximum number of hits obtained with *B. mori* can be attributed to the higher number of sequences in the database as compared to other insect species (Figure S2).

**Figure 3:**
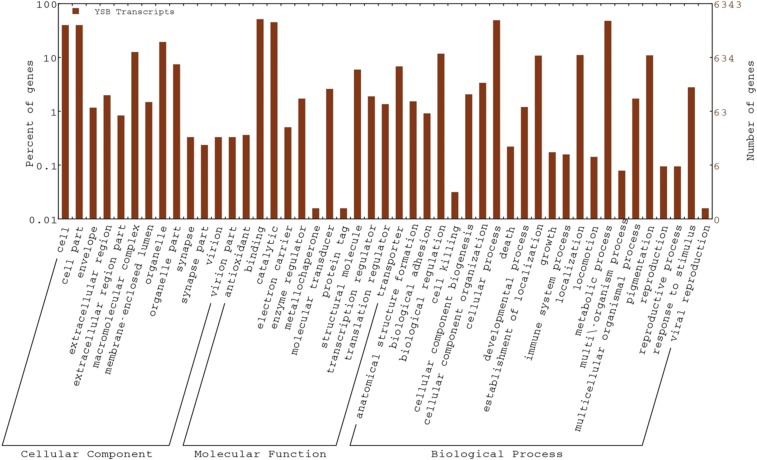
Gene Ontology classification of *S. incertulas* transcripts. YSB transcripts were classified into biological process, cellular component, and molecular function. The left and right *y*-axes denote separately the percent and number of genes in the particular category. YSB, yellow stem borer.

**Figure 4:**
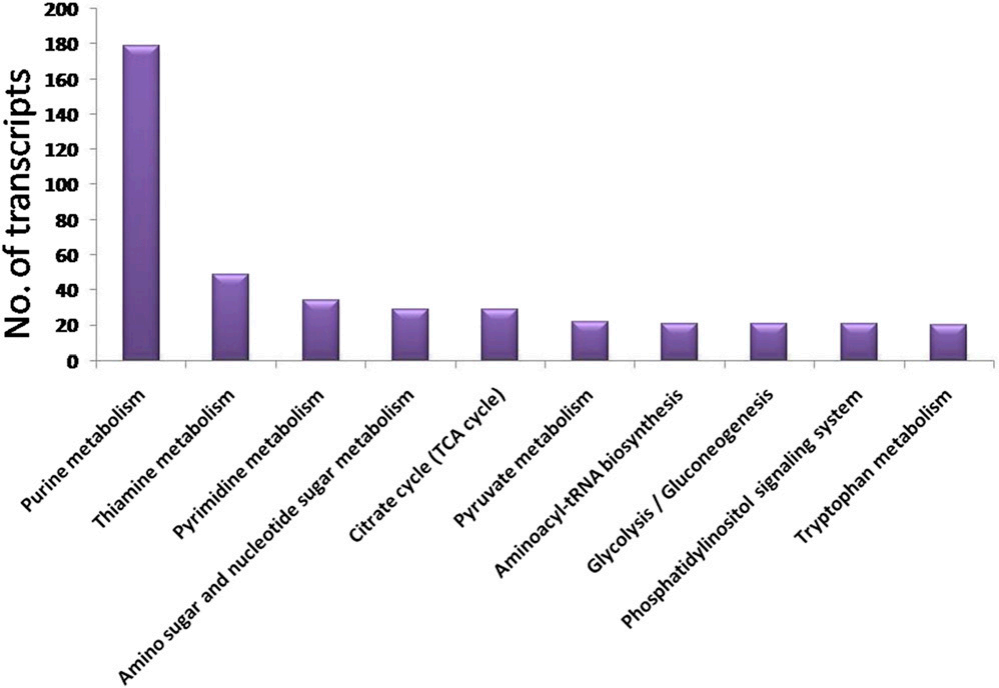
Top 10 metabolic pathways represented in *S. incertulas* transcriptome through KEGG pathway analysis. KEGG, Kyoto Encyclopedia of Genes and Genomes; TCA, tricarboxylic acid cycle.

Based on GO, the YSB transcripts were divided into three major categories: cellular component, molecular function, and biological processes. In the cellular component category, the highest number of transcripts were attributed to cell (1998, GO: 0005623) and cellular part (1998; GO: 0044464). In the molecular function category, the maximum number of transcripts were attributed to binding (2569; GO: 0005488) followed by catalytic function (2392; GO: 0003824). In the biological process category, the maximum number of transcripts were associated with cellular process (2449; GO: 0009987) followed by metabolic process (2395; GO: 0008152) (Figure 3). The metabolic pathway enrichment through KEGG confronted a total of 1078 transcripts, which were mapped to 88 metabolic pathways, and the top ranking 10 metabolic pathways are shown in Figure 4. The highest number of transcripts was mapped to purine metabolism (179, 16.6%). It is the most critical pathway for the generation of energy sources like ATPs and GTPs, necessary for cellular functions in all organisms. Purine metabolism is also involved in transcriptional regulation during sex-specific nutrition allocation in *Drosophila melanogaster* which influences reproduction, repair, and aging processes (Bauer *et al.* 2006).

From the total transcriptome, the transcripts associated with five important classes were selected for further discussion, namely insecticide detoxification and target enzymes, chemoreception for host specificity, RNAi machinery, TFs, hormonal biosynthesis, and visual perception.

### Insecticide detoxification and target enzymes

Insecticides such as pyrethroids and neonicotinoids show high toxicity to insect pests, but relative hypotoxicity to mammals and natural enemies. One of the main reasons for this phenomenon is variation attributed to the presence of insecticide target genes and their metabolism (Li *et al.* 2007). In YSB, genetic information on insecticide resistance has yet not been reported. We identified transcripts related to insecticide detoxification like cytochrome P450 (CYP450), glutathione S-transferases (GSTs), and CarEs. In addition, insecticide targets like acetylcholinesterase (AChE), γ-aminobutyric acid receptor (GABA), nicotinic acetylcholine receptor (nAChR), sodium channels, and ligand-gated chloride channels were also found (Table S4).

**Figure 5:**
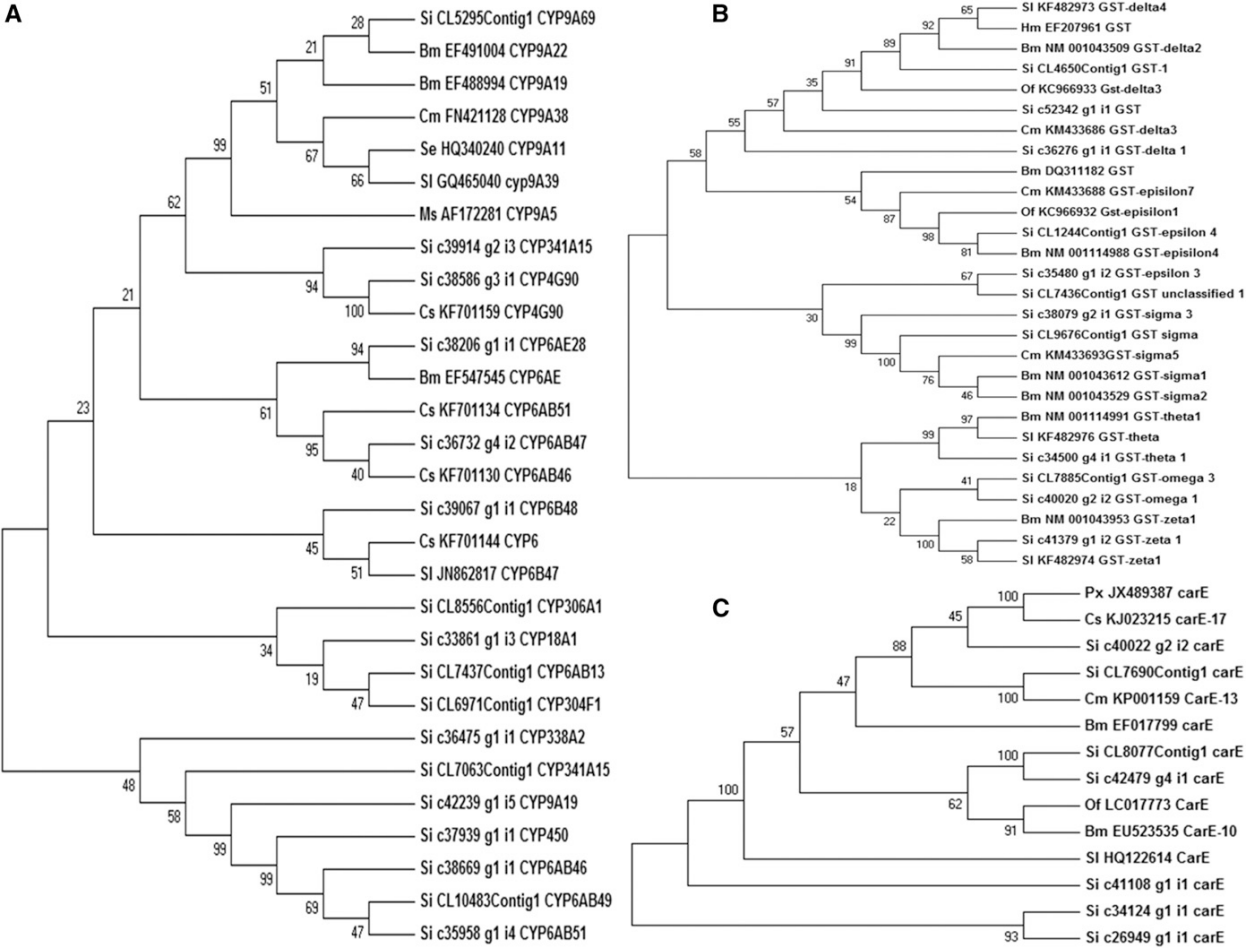
Phylogenetic tree of *S. incertulas* transcripts encoding insecticide mechanism along with other lepidopteran insects. (a) Cytochrome P450 (CYP450); (b) Glutathione S-transferases (GSTs); and (c) Carboxylesterases (CarEs). The tree was constructed from the multiple alignments using MEGA 5.0 software and generated with 1000 bootstrap trials using the Neighbor-Joining method. The numbers at the top of each node indicate bootstrap confidence values obtained for each node after 1000 repetitions. Bm, *B. mori*; Cm, *C. medinalis*; Cs, *C. suppressalis*; Hm, *Heliconius melpomene*; Ms, *M. sexta*; Of, *O. furnacalis*; Px, *Plutella xylostella*; Se, *S. exigua*; Si, *S. incertulas*; Sl, *S. litura*.

CYP450 is one of the largest protein super families in insect species, functioning in xenobiotic metabolism and detoxification (Scott 2008). Among the 80 CYP450 transcripts identified in this study, 17 transcripts showing > 75% similarity in the nr database were used for phylogenetic analysis. The majority of the CYP450 family transcripts were unique to YSB, except a few that clustered with other insects such as *C. suppressalis* (Figure 5a). CYP450 has three important families, namely CYP4, CYP6, and CYP9. The CYP4 genes in insects are involved in both pesticide metabolism and chemical communication. Previous studies have demonstrated that the CYP4 family genes CYP4C27 in *Anopheles gambiae* (David *et al.* 2005) and CYP4G19 in *Blattella germanica* (Pridgeon *et al.* 2003) are associated with insecticide resistance. Similarly, CYP6B48 and CYP6B58 in *Sp. litura* (Wang *et al.* 2015), CYP6AE14 and CYP6AE12 in *H. armigera* (Zhou *et al.* 2010), and CYP9A2 in *Manduca sexta* (Stevens *et al.* 2000) were reported for their expression in response to plant allelo chemicals and xenobiotics. In the current study, we have reported the presence of common and unique CYP genes in YSB for the first time, which can be further characterized and exploited.

The GTSs belong to a family of multifunctional detoxification enzymes that catalyze the conjugation of reduced glutathione with exogenous and endogenous toxic compounds or their metabolites. In insects, GSTs mediate resistance to organophosphates (OPs), organochlorines, and pyrethroids (Yamamoto *et al.* 2009). The main classes of GSTs in insects are delta, epsilon, omega, sigma, theta, zeta and microsomal. The delta and epsilon classes comprise the largest insect-specific group of GST classes and play an important role in xenobiotic detoxification (Ranson *et al.* 2002). They are well-studied in insect species like *Lucilia cuprina*, *N. lugens*, *P. xylostella*, *M. sexta*, *Musca domestica* (Enayati *et al.* 2005), and *B. mori* (Yamamoto *et al.* 2009). An epsilon GST was reported to detoxify DDT resulting in DDT-resistance in *A. gambiae* (Ortelli *et al.* 2003), while a delta class GST showed induced resistance to pyrethroids by detoxifying lipid peroxidation products (Vontas *et al.* 2001). We identified a total of 23 transcripts encoding GSTs. Twelve transcripts of GST (representing delta, epsilon, zeta, sigma and omega classes) with > 75% similarity in the nr database and an average length of 1518 bp were considered for phylogenetic analysis. The GST transcripts of YSB shared considerable similarity with *B. mori* followed by *O. furnacalis* (Figure 5b). Here also, common and unique transcripts related to GSTs in YSB were identified, which can be later characterized for future development of effective insecticides.

Carboxylesterases are enzymes of the carboxyl/cholinesterase gene family that catalyze the hydrolysis of carboxylic esters to their component alcohols and acids. They have several physiological functions, such as the degradation of neurotransmitters and metabolism of specific hormones and pheromones (Cui *et al.* 2011). Carboxylesterases offer insect resistance to OPs, carabamates, and pyrethroids by gene amplification or transcriptional upregulation (Yan *et al.* 2009). Carboxylesterases have been extensively studied in insects like *L. cuprina* and *D. melanogaster* (Heidari *et al.* 2005). Here, seven CarE transcripts with an average length of 2377 bp were used for phylogenetic tree construction, which showed considerable similarity with CarE of *Cn. medinalis* (Figure 5c). Although the molecular mechanism of insecticide action is still unknown in YSB, the present transcriptome study provides baseline information on insecticide target enzymes and genes associated with insecticide detoxification. These results indicate that YSB has transcripts that are involved in detoxification mechanisms, so these may be the potential targets for suppression to bring down insecticide resistance in YSB (Kola *et al.* 2015).

### Chemoreception for host specificity

In insects, both larvae and adults use their olfactory system to detect food resources, sexual partners, or adequate oviposition sites. Chemoreception occurs by recognition of plant volatiles and uptake of chemical components from the environment and their interaction with chemoreceptors (Smith and Getz 1994). Generally, the chemoreception system in insects has important components, namely odorant binding proteins (OBPs), chemosensory proteins (CSPs), odorant receptors (ORs), sensory neuron membrane proteins, ionotropic receptors (IRs), and gustatory receptors (GRs) (Leal 2013; Jia *et al.* 2016). The OBPs and CSPs are signal binding proteins that recognize chemical cues from the ambient environment, whereas ORs, IRs, and GRs convert chemical signals into neuronal activity and thus play key roles in local adaptation and reproductive isolation in insects. The diversity of ORs between and within the insect species imparts specificity to insects and allows these receptors to bind to a variety of ligands (Sato *et al.* 2008). Genome sequencing and molecular studies have characterized the complete OR repertoires and other olfactory genes in several insect species such as *B. mori* (Gong *et al.* 2009) and *Conogethes punctiferalis* (Jia *et al.* 2016), providing an understanding of the olfactory signal pathways in these insects. In the YSB transcriptome, a total of 62 transcripts related to chemoreception were identified. Among these transcripts, 21 transcripts including four CSPs, three GRs, nine OBPs, and five ORs were selected for phylogenetic analysis to determine their relationship with other insect species (Table S4).

**Figure 6:**
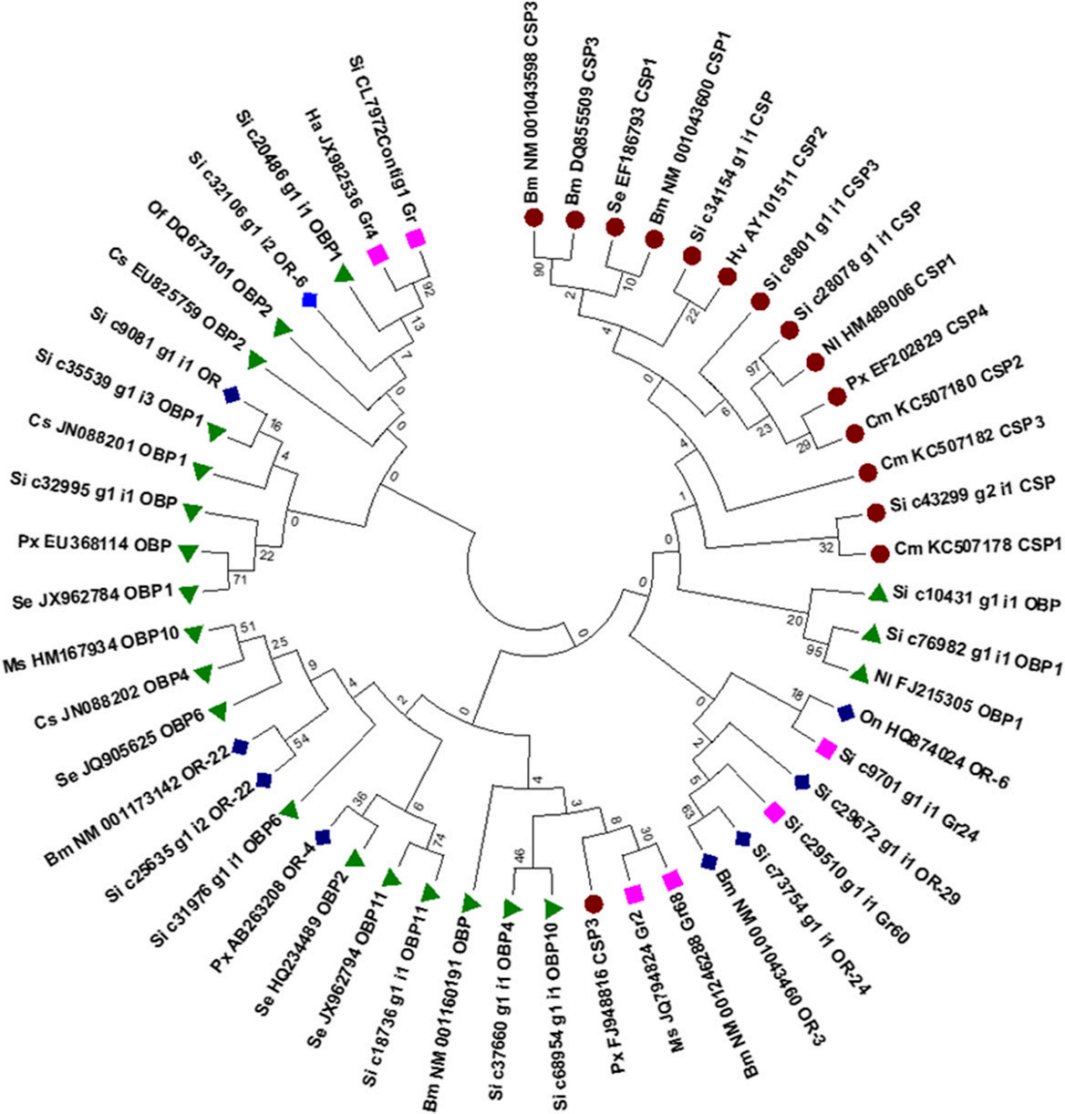
Phylogenetic tree for annotated sequences of chemoreception mechanism among *S. incertulas* (Si), lepidopteran, and hemipteran insects. Bm, *B. mori*; Cm, *Cn. medinalis*; Cs, *C. suppressalis*; Ha, *H. armigera*; Hv, *He. virescens*; Ms, *M. sexta*; Nl, *N. lugens*; On, *O. nubilalis*; Of, *O. furnacalis*; Px, *P. xylostella*; Se, *S. exigua*;.

YSB transcripts coding for OBP (si c76982g1i1 OBP1) and CSP (si c8801g1i1CSP) shared high homology with the rice insect *N. lugens* and showed high bootstrap values (Figure 6). Similar observations were made with protein sequence comparison (99% homology). These two transcripts matched those of another monophagous rice insect, indicating that they may be crucial for rice recognition by insects. The remaining chemoreception associated transcripts of YSB did not show any homology with other insects, suggesting that YSB has independently evolved its chemoreception machinery leading to stenophagy. The finding that the two transcripts are common in two specialist feeders of rice; namely YSB and BPH is worth probing further, to ascertain their role in host finding. Although the function of OBP, GRs, ORs, and the host specificity mechanism in YSB are not clear, earlier studies in other insects have suggested their tight association with plant volatile reception (Xue *et al.* 2014). Through RNA-seq, chemosensory genes have been identified and their significance in host recognition was reported in some lepidopteran species like *H. armigera* (Liu *et al.* 2012), *Cydia pomonella* (Bengtsson *et al.* 2012), and *M. sexta* (Grosse-Wilde *et al.* 2011). The presence of an olfactory system in another lepidopteran pest, cotton leaf worm (*S. littoralis*), also gives clues regarding the chemosensory behaviors of insects. The present study confirms the existence of a robust chemoreception mechanism in YSB via the presence of all the other important components required for chemoreception, which might be responsible for host detection and the monophagous nature of this insect.

### RNAi machinery

The existence of RNAi machinery in animals, plants, and fungi has enabled it to be applied to gene knockdown studies more efficiently. In insects, RNAi is triggered by the presence of dsRNA or siRNAs (Huvenne and Smagghe 2012). Although, RNAi mechanisms exist in some insects, the differences regarding signal amplification, systemic effect, and inheritance vary among the species (Posnien *et al.* 2009). Dicer1 and Dicer2 are known to cleave the long dsRNA into siRNAs (∼21–25 nt). The presence of dicer genes was reported in *T. castaneum* (*Tc-Dcr-1* and *Tc-Dcr-2*) and *S. litura* (*Dcr1 and Dcr2*) (Tomoyasu *et al.* 2008; Gong *et al.* 2015). Argonaute (Ago) proteins bind to single-stranded mRNA through siRNAs to degrade it in a sequence-specific manner (Hammond *et al.* 2001). The presence of two *Ago* genes in *N. lugens*, *Ago1* and *Ago2* in *S. litura*, and five types of *Ago* in *T. castaneum* (*Tc-ago-1*, *Tc-ago-2a*, *Tc-ago-2b*, *Tc-ago-3*, and *Tc-Piwi*) have been reported (Tomoyasu *et al.* 2008; Xu *et al.* 2013; Gong *et al.* 2015). Sid proteins are well-known for the uptake and spread of gene silencing signals. The presence of these proteins has been reported in *Apis mellifera* (Aronstein *et al.* 2006), *B. mori* (Kobayashi *et al.* 2012), and *S. litura* (Gong *et al.* 2015).

**Figure 7:**
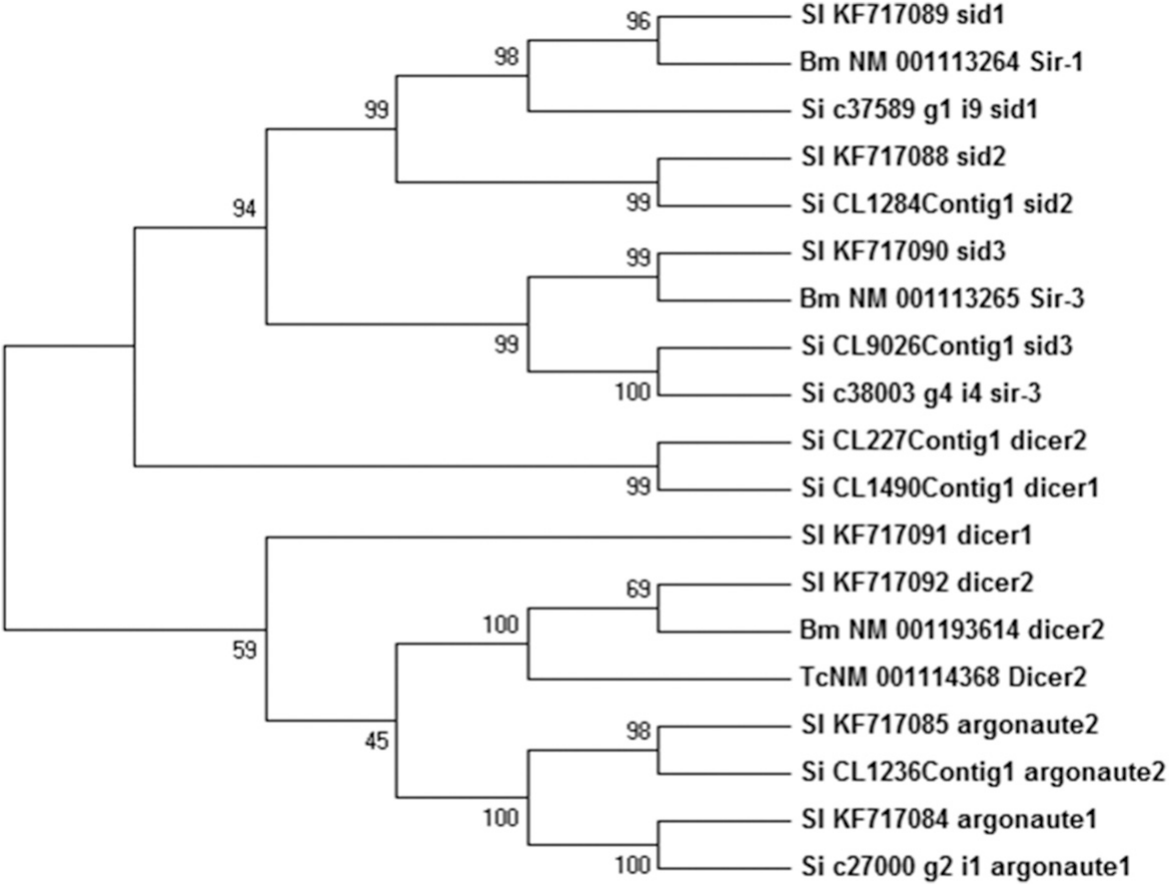
Phylogenetic tree of RNA interference machinery from *S. incertulas* (Si) with lepidopteran and coleopteran insects. Bm, *B. mori*; Sl, *S. litura*; Tc, *T*. *castenum*.

In YSB, core genes of RNAi machinery, namely Dicer1 (*Dcr-1*), Dicer2 (*Dcr-2*), Argonaute1 (*Ago-1*), Argonaute2 (*Ago-2*), *Sid-1*, *Sid-2*, *Sid-3*, and *Sid-1*-related gene were found that indicates that YSB has complete RNAi machinery (Table S4). Until now, evidence to support the existence of RNAi machinery in YSB has not been reported. Thus, these findings are important in that they open up the possibility of exploring RNAi strategies for the management of YSB. In our previous study, we have shown the silencing of genes, *i.e.*, Aminopeptidase (APN) and CYP450 (CYP6) by feeding the YSB larvae with dsRNAs (Kola *et al.* 2016). Through transcriptome analysis, the presence of RNAi genes and a silencing response have been reported in *Cylas puncticollis* (Prentice *et al.* 2015) and *Tuta absoluta* (Camargo *et al.* 2015). It is interesting to note that the YSB transcripts encoding *Sid-1*, *Sid-2*, *Ago-1*, and *Ago-2* revealed higher homology with another lepidopteran pest, *S. litura*, in which RNAi has been exploited very well for its control (Figure 7).

### Transcription factors

Gene regulation can be controlled by DNA- and protein-binding TFs at both the transcriptional and translational level. A total of 415 TFs were identified in the YSB transcriptome, of which the majority of the TFs belongs to zinc fingers (ZFs) (400) followed by Myb proteins (8). The Cys_2_His_2_ ZFs are the most common DNA-binding motifs in all eukaryotes (Wolfe *et al.* 2000). It has been well-established that ZF TF’s are involved in DNA recognition, RNA packaging, transcriptional activation, and lipid binding, which is required for growth and development in both animals and plants (Laity *et al.* 2001). In red beetle (*Tribolium*), Sp-like ZF TF (T-Sp8) was reported to be involved in formation of body appendages and the regulation of growth (Beermann *et al.* 2004). The other TFs, namely leucine-zipper TFs, basic helix-loop-helix (bHLH) TFs, and β-NAC-like protein were less in number. The bHLH TFs are also known to play important roles in controlling cell proliferation and tissue differentiation (Kageyama and Nakanishi 1997), and the developmental processes of *D. melanogaster* (Moore *et al.* 2000) and *B. mori* (Zhao *et al.* 2014).

### Hormone biosynthesis

In insects, development, moulting, and other related processes are regulated by juvenile hormone (JH). Degradation of JH occurs mainly by the action of juvenile hormone esterase (JHE) and/or juvenile hormone epoxide hydrolase (JHEH) (Belles *et al.* 2005). In the YSB transcriptome, we also found the presence of JHE, JHEH, and JH-binding protein. Another important insect hormone, prothoracicotropic hormone (PTTH), stimulates the secretion of moulting hormone (ecdysone) from prothoracic glands and regulates the developmental timings in insects (McBrayer *et al.* 2007). The presence of this hormone (YSB_LS_c64818_g1_i1) was also evidenced in our work. Insect ecdysis behavior at each developmental stage will be determined by ecdysis-triggering hormone (Park *et al.* 2003). In YSB, we also found the presence of ecdysone receptors (YSB_LS_c42438_g1_i1 and YSB_LS_CL10106Contig1), which can be used as selective insecticides. Earlier studies have also reported that ecdysone receptor (EcR) has been used as a potential target for RNAi based pest control in *H. armigera* (Zhu *et al.* 2012) and *N. lugens* (Yu *et al.* 2014).

### Visual perception

The r-opsin family of proteins is instrumental in visual perception and host recognition in arthropods (Feuda *et al.* 2016). In YSB, we also found transcripts encoding long-wavelength opsin (YSB_LS_CL7470Contig1), UV opsin (YSB_LS_c32301_g1_i1), and blue opsin (YSB_LS_c33970_g1_i1) in all the four larval developmental stages. In addition, rhodopsins like Rh_1_ and Rh_3_ were also found at different developmental stages, which may indicate that they have an important role in host recognition apart from chemoreception.

### Differential gene expression

**Figure 8:**
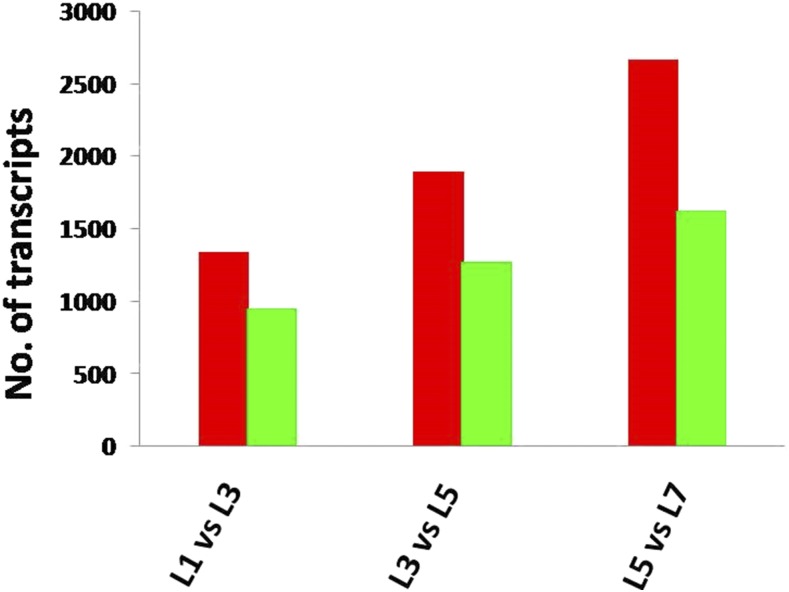
Differentially expressed transcripts between four larval developmental stages of *S. incertulas*. Up- (red) and downregulated (green) YSB transcripts between the four developmental stages. L_1_, first instar larvae; L_3_, third instar larvae; L_5_, fifth instar larvae; L_7_, seventh instar larvae; YSB, yellow stem borer.

To determine the level of gene expression during YSB larval developmental stages, transcripts were analyzed on the basis of FPKM values. Overall, 9775 transcripts were differentially expressed across the four stages of larval development (Figure 8). In L_3_ compared to L_1_, 1340 and 951 transcripts were up- and downregulated. Transcripts exclusively expressed at L_1_ and L_3_ were 388 and 206, respectively; 1697 transcripts were common to both stages (Table S5). In the top 10 differentially upregulated transcripts, most were associated with cuticular proteins, suggesting that cuticle formation may be the key process at this stage of larval development. Cuticular proteins and chitin are essentially required to maintain physical structure and localized mechanical activities (Andersen *et al.* 1995). Thus, these cuticular proteins might also be involved in maintaining physical structure in YSB.

A list of the top 10 up- and downregulated transcripts is given in Table S6. Interestingly, the transcript encoding long-wave opsin (YSB_LS_c24494_g1) was highly expressed only at the L_1_ stage compared to other stages. It is required for visual perception according to environmental cues and has a prominent role in insect behavior, as reported in *H. armigera* (Yan *et al.* 2014). This could be of significance in YSB, as it may aid in dispersal of the just-hatched larvae and their ability to find the host plant. In addition to the opsin, one of the neuropeptide precursors (YSB_LS_c24589_g1) was also highly expressed at the L_1_ stage compared to other larval stages. The roles of neuropeptides and their precursors in development, physiology, and behavior were previously reported in several insects like *N. lugens* (Tanaka *et al.* 2014) and *T. castaneum* (Li *et al.* 2008).

Similarly, between L_3_ and L_5_, 3172 differentially expressed transcripts were observed, of which 1896 were upregulated and 1276 were downregulated at L5. Three hundred and sixty-eight transcripts at L_3_ stage and 314 transcripts at L_5_ stage were expressed exclusively, whereas 2490 transcripts were expressed commonly in both stages. Among the top 10 differentially upregulated transcripts, two transcripts; discs large1 and Aminopeptidase N3 (APN3) are noteworthy. Hexamerine, reverse transcriptase, and β-fructofuranosidase 2 transcripts were downregulated. The APN has an important role in dietary protein digestion in the midgut (Wang *et al.* 2005). It can be postulated that the APN gene in YSB may also have a prominent role in the larval midgut and can be used as a candidate for gene silencing. Previous studies also demonstrated that inhibition of its activity in the larval midgut can result in a detrimental effect on larval growth and development, finally leading to larval death (Ningshen *et al.* 2013). At the L_3_ stage, the maximum number of transcripts involved in insecticide detoxification was expressed. EcR, nAChR, and GABA were more highly expressed at the L_3_ stage than at all other stages, which indicates that molting and neurotransmission are key processes at this stage of development. The larval developmental transitions (molting and metamorphosis) are largely regulated by changes in the titer of the ecdysteroid hormone 20-hydroxyecdysone (20E) binding to its EcR. The function of EcR isoform-A (BgEcR-A) has been reported through RNAi in *Bl*. *germanica* (Cruz *et al.* 2006). Insect nAChR and GABA receptors mediate postsynaptic cholinergic transmission (Raymond-Delpech *et al.* 2005). The effect of nAChR on the insect central nervous system has been previously reported in *A. mellifera* (Jones *et al.* 2006) and *B. mori* (Shao *et al.* 2007).

**Figure 9:**
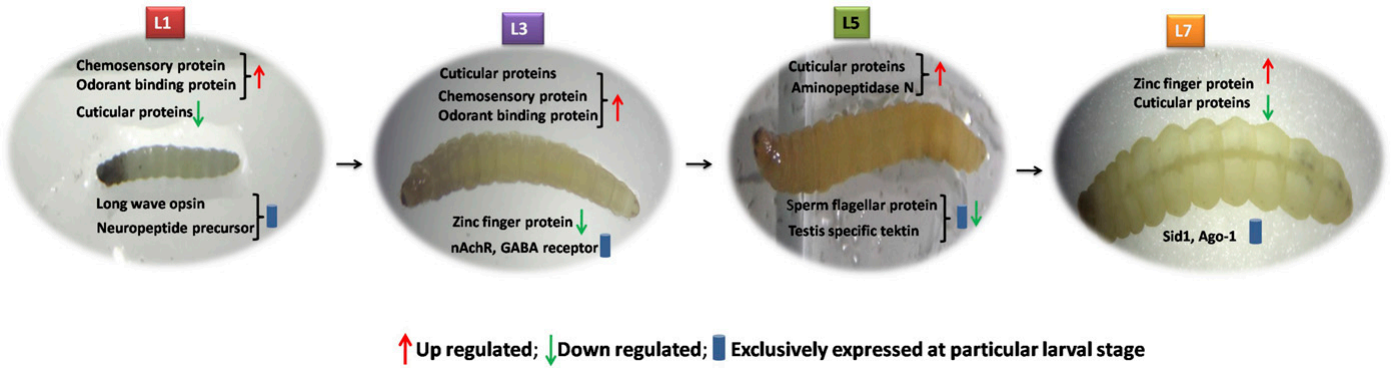
Significantly up- and downregulated and exclusively expressed transcripts at each stage of *S. incertulas* larval development.

Between L_5_ and L_7_, 4292 transcripts were differentially expressed, of which 2668 were upregulated and 1624 were downregulated at L_7_. Of these, 3405 transcripts were expressed in both the stages, while 463 and 424 transcripts were exclusively expressed at L_3_ and L_5_, respectively. Among the top 10 differentially upregulated transcripts, ZF protein 600 (ZFP600) and CarE were highly upregulated while the maximum transcripts encoding cuticular proteins were downregulated. The sperm flagellar protein (YSB_LS_c17944_g1) and testis-specific tektin (YSB_LS_c7891_g1) were exclusively highly expressed at the L_5_ stage compared to all other stages, which indicates that these transcripts could be involved in sperm formation and maturation, particularly during this instar of YSB. The role of testis-specific protein (BmTst) for spermatogenesis has been reported in *B. mori* (Ota *et al.* 2002), but in YSB, no information is available so far on spermatogenesis. The role of tetkin in YSB need to be probed further to understand the mechanism. Among exclusively expressed transcripts at the L_7_ stage, the majority were TFs. Components of the RNAi gene silencing machinery like *Sid1* (YSB_LS_c38003_g4) and *Ago-1* (YSB_LS_c27000_g2) were also highly expressed only at L_7._ The transcripts for ZF proteins, CarEs, cuticular proteins, and voltage-gated sodium channels were also highly expressed at the L_7_ stage, and these may be used as potential candidate genes for gene silencing through RNAi. The number of differentially expressed transcripts increased during the larval transitions from L_1_ to L_7_. This indicates that L_7_ might be the most evolved stage of YSB development proceeding to the pupal stage. These transcripts might be involved in metamorphosis and modulated by the expression of tissue-specific transcripts. The significantly up- and downregulated and exclusively expressed transcripts at each stage of larval development are represented in Figure 9. JH hormone regulation is usually controlled by JHE, JHEH, and JH-binding protein. The expression of JHE (YSB_LS_CL6858Contig1) and JHEH (YSB_LS_CL2874Contig1) decreased gradually from L_1_ to L_7_, while that of JH-binding protein was significantly decreased at the L_7_ stage, indicating that JH regulation is crucial for YSB development. During unfavorable conditions, YSB remains as larva and may undergo up to 10 larval moults with low levels of JH-binding protein. In general, a lack of PTTH results in delayed larval development and eclosion (McBrayer *et al.* 2007). Inhibition of PTTH has been shown to regulate the larval diapauses (Chippendale 1977). Interestingly, low expression of PTTH (YSB_LS_c64818_g1_i1) was observed at the L_7_ stage, suggesting that PTTH may have a role in YSB development.

**Figure 10:**
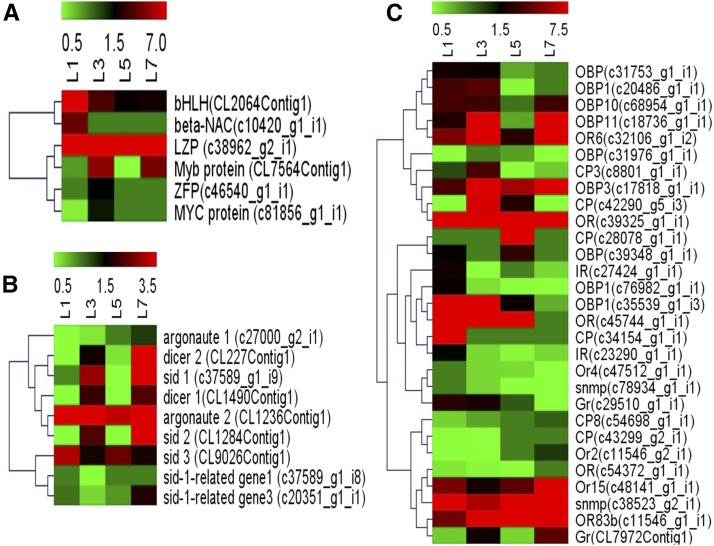
Expression analysis of YSB transcripts at four developmental stages based on their relative FPKM values. Transcripts were hierarchically cluster based on average Pearson distance, complete linkage method. (a) Transcripts in major TF families; (b) Transcripts in RNAi machinery; and (c) Transcripts in chemoreception. Green indicates the lowest level of expression, black indicates the intermediate level of expression, and red indicates the highest level of expression. FPKM, Fragments Per Kilobase of transcript per Million mapped reads; L_1_, first instar larvae; L_3_, third instar larvae; L_5_, fifth instar larvae; L_7_, seventh instar larvae; RNAi, RNA interference; TF, transcription factor; YSB, yellow stem borer.

Comparing all the four YSB larval developmental stages, the maximum number of commonly expressed transcripts was observed for cuticle proteins, chymotrypsins, CYP450s, GSTs, APN, CarE, ZFPs, CSPs, and OBPs. The consistent expression of these transcripts provides evidence for their role in insect growth and development. The functionally important transcripts belonging to the RNAi machinery, chemoreception, and TFs were analyzed based on their normalized gene expression values (FPKM) and represented as heat maps based on the hierarchical clustering of transcripts (Figure 10). In the case of TFs, the transcript for leucine-zipper protein was expressed continuously in all larval stages, indicating that it has an important functional role in the regulation of gene expression during larval stages. However, one of the transcripts encoding β-NAC was expressed only at the L_1_ stage. In the case of RNAi machinery, *Ago-2* was highly expressed in all larval stages but *Ago-1* showed reduced expression, which indicated that *Ago-2* might be more involved in the RNAi pathway in YSB. In contrast, at the L_7_ stage, *dcr-2*, *sid-1*, and *sid-2* were highly expressed, which further shows that the RNAi mechanism might be functional at the L_7_ stage. The expression of chemoreception-associated transcripts in YSB was the highest at the L_1_ and L_3_ larval stages; however, transcripts for ORs were constitutively expressed in all larval stages, indicating that they might play a crucial role in host specificity. Pairwise comparisons between stages on the basis of fold change and false discovery rate between the samples have been depicted as MA and Volcano plots (Figure S3).

### qRT-PCR validation

**Figure 11:**
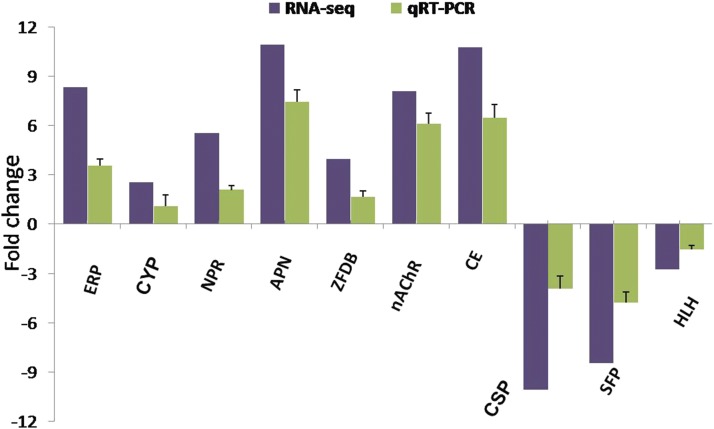
Validation of the RNA-sequencing (RNA-seq) data using quantitative reverse transcription-polymerase chain reaction (qRT-PCR). All data were normalized to the reference gene, yellow stem borer β-actin. The transcripts validated were ecdysteroid-regulated protein (ERP), cytochrome p450 (CYP6AB51), neuropeptide receptor A10 (NPR) aminopeptidase N (APN), zinc finger DNA-binding protein (ZFDB), nicotinic acetylcholine receptor (nAChR), carboxylesterase (CarE), chemosensory protein (CSP), seminal fluid protein (SFP), and helix-loop-helix protein (HLH). Error bars indicates statistical significance of data.

We selected 10 transcripts based on their molecular function, namely reproduction, insecticidal detoxification, protein digestion, molting, and chemoreception, for validation through qRT-PCR. These transcripts are: ecdysteroid-regulated protein, CYP450 (CYP6AB51), neuropeptide receptor A10, APN, ZF DNA-binding protein, nAChR, CarE, chemosensory protein (CSP), seminal fluid protein (SFP), and helix-loop-helix protein (HLH). All selected transcripts were amplified by qRT-PCR and showed significant differential expression. The results obtained in qRT-PCR were positively correlated (*R*^2^ = 0.96) with RNA-seq data (Figure 11).

### YSB EST-SSR mining and characterization

SSR mining identified a total of 1327 (5%) transcripts harboring SSR motifs. The newly developed SSRs with regard to the transcripts derived from RNA-seq were referred to as YSB EST-SSR. We categorized the SSR motifs into perfect SSRs (repeat motifs that are simple tandem sequences, without any interruptions) and compound SSRs (sequences containing two adjacent distinct SSRs, separated by none to any number of base pairs) (Parekh *et al.* 2016). In our study, we kept this distance as 100 bp and excluded mononucleotide repeats to reduce the sequencing errors in the data (Kalia *et al.* 2011). A total of 563 SSR motifs were found in the YSB transcriptome with a frequency of one SSR/10.98 kb (Table 3). Comparatively, the frequency of YSB EST-SSRs was higher than in earlier reports in insect species (Hamarsheh and Amro 2011). This further strengthens the good transcriptome coverage achieved by RNA-seq. Out of the 563 SSR motifs, 511 SSR motifs belonged to class І (≥ 20 bp) and 52 to class II (< 20 bp) (Table 4). Considering the reverse and complementary sequences and/or different reading frames, (AC)*n*, (CA)*n*, (TG)*n*, and (GT)*n* were counted as the same class (Yang *et al.* 2012). On the basis of identified SSRs, trinucleotide repeats were the most represented (59.5%), with motif repeats CCG/CGG, followed by dinucleotide repeats with 35.1% motifs of AT/AT (Table S7). It is a fact that frameshift mutations or additions/deletions in trinucleotide repeats do not disturb the open reading frame (Metzgar *et al.* 2000). This further supports the highest number of trinucleotide repeats obtained in the present study. The repeat type AT/AT was also found to be the highest in the Asian giant hornet *V. mandarinia* (Patnaik *et al.* 2016). The EST-SSRs are worthy genetic resources for understanding genetic diversity, and are comparatively more efficient than genomic SSRs in population genetic studies (Arias *et al.* 2011). The YSB EST-SSRs provide a valuable resource for future genetic analysis of *S. incertulas* and can be employed for constructing high-density linkage maps of YSB. Moreover, YSB EST-SSRs can be used for cross-species/taxa identification, which can also yield preliminary information regarding other related but unexplored insect taxa (Stolle *et al.* 2013).

**Table 3:** Statistics of SSRs identified in *S. incertulas* transcripts

Parameter	Value
Total no. of sequences examined	24,773
Total size of examined sequences (bp)	14,563,840
Total no. of identified SSRs	1,327
No. of SSR-containing sequences	1,239 (5%)
No. of sequences containing > 1 SSR	80
No. of SSRs present in compound formation	45

Frequency of SSRs one per 10.98 kb. SSR, simple-sequence repeats.

**Table 4:** Classification of YSB EST-SSR according to motif length

Motif Length	Class I	Class II	Total No. of SSR	Avg. Frequency
	(≥ 20 bp)	(≥ 10 but < 20 bp)		(kb/SSR)
Dinucleotide	193	5	198	185.75
Trinucleotide	297	38	335	109.78
Tetranucleotide	21	4	25	1,471.18
Pentanucleotide	0	4	4	9,194.90
Hexanucleotide	0	1	1	36,779.61
Total	511	52	563	

No., number; SSR, simple-sequence repeats; Avg., average.

### Comparative transcriptome analysis

**Figure 12:**
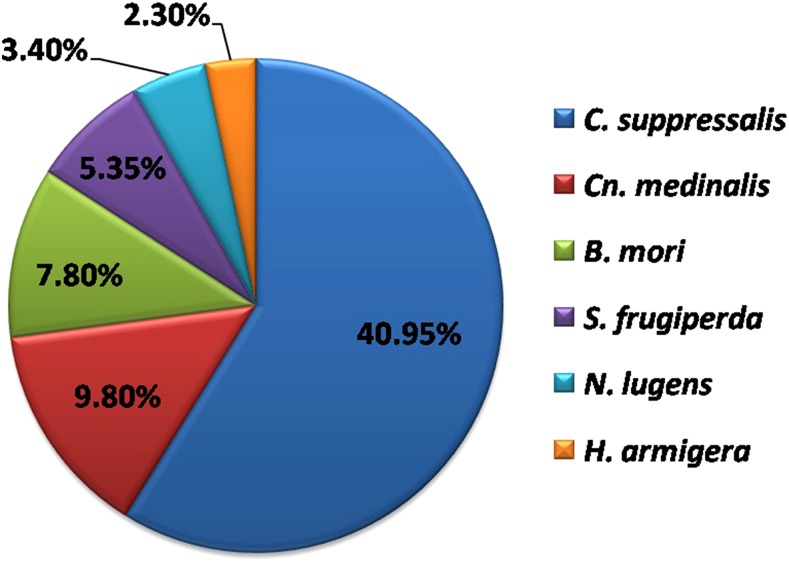
Comparative analysis of yellow stem borer transcriptome with lepidopteran and hemipteran insect protein sequences at a cut-off *E*-value 10^−6^ and ≤ 80% similarity.

To understand the similarity or variability among different insect species on the basis of sequence conservation, comparative transcriptome analysis of YSB was done with six related insect species. It showed significant homology with *C. suppressalis* (4186) followed by *N. lugens* (3787), *B. mori* (3027), *S. frugiperda* (452), *H. armigera* (293), and *Cn. medinalis* (27) (Figure 12). We found that most of the transcript (63) sequences are unique to YSB on the basis of less percentage identity with related species. Transcripts related to detoxification mechanisms, namely nAChR (YSB_LS_CL9841Contig1), AChE (YSB_LS_c43640_g1_i3), GSTs (YSB_LS_c41379_g1_i2), as well as the voltage-gated sodium channel (YSB_LS_CL609Contig1) and GABA receptor (YSB_LS_c20927_g2_i1), showed 95–100% similarity with *C. suppressalis* and *Cn. medinalis*, which are predominant rice insects in South Asian countries. Based on this observation, it appears that the detoxification mechanism is conserved across the lepidopteran rice pests, So these genes could be explored for silencing through RNAi to achieve broad-spectrum resistance in rice (He *et al.* 2012). In contrast, two of the transcripts encoding the voltage-gated ion channel and CYP450 showed ≤ 20% similarity with all other pests, suggesting that they are specific to YSB. In the RNAi machinery, the transcript for *Sid-2* (YSB_LS_CL1284Contig1) and *Ago-1* (YSB_LS_c27000_g2_i1) showed 95% sequence homology with *C. suppressalis* and *N. lugens*. However, *Ago-2*, *Sid-1*, *Sid-3*, and *sid-1*-related genes showed less homology (40–50%), suggesting that the evolution of the RNAi mechanism may be driven by the host. Interestingly, the transcript responsible for visual recognition, *i.e.*, long wave opsin (YSB_LS_c24494_g1_i1), and transcripts involved in the chemoreception mechanism (CSP and OBP) showed 98–100% homology with BPH. Notably, BPH and YSB have only rice as a host and both are monophagous insects. From the homology of host-specific recognition transcripts, it can be postulated that these transcripts might be playing an important role in the monophagy of YSB and BPH in rice. Similarly, the transcripts common to both *C. suppressalis* and YSB suggested an association with feeding behavior, as both are borers on rice crops. Generally, the morphology of mouthparts plays a major role in the boring, chewing, or sucking capability of insects (Rogers *et al.* 2002), but tremendous diversification has been reported in insect mouthparts corresponding to their food niches. In YSB, transcripts like sex combs reduced (scr) homolog (YSB_LS_c35525_g1_i2), Hox protein pb (YSB_LS_c642_g2_i1), and distal-less-like (dl) protein (YSB_LS_c27814_g1_i2) were identified, which showed higher similarity (≤ 80%) with *B. mori* and *C. suppressalis* (chewing type of mouth parts) and have less similarity (≥ 52%) with *N. lugens* (sucking pest). This implies that these three transcripts may be required for the feeding and boring ability of YSB. Nevertheless, further in-depth research is needed for better understanding of the feeding behavior of YSB.

Among 63 YSB-specific transcripts, most coded for unpredicted (7), putative (3), and hypothetical proteins (13). This could be due to uniqueness of transcripts for YSB, which has not been very well-studied at the molecular level. The remaining transcripts, whose functions are known, includes adipokinetic hormone receptor (YSB_LS_c40297_g7_i2), CarE (YSB_LS_c41108_g1_i1), muscle M-line assembly protein (YSB_LS_c39945_g2_i1), WD repeat domain-containing protein (YSB_LS_CL8220Contig1), ZF protein (YSB_LS_c40816_g4_i5), allantoinase (YSB_LS_c37251_g1_i6), neuropeptide receptor (YSB_LS_c32700_g1_i1), leucine-zipper TF (YSB_LS_CL2667Contig), CYP450 (YSB_LS_c43088_g1_i5), titin-like protein (YSB_LS_c42866_g3_i1), U5 small nuclear ribonucleoprotein (YSB_LS_c10666_g1_i1), mitochondrial carrier protein (YSB_LS_c39431_g4_i1), and reverse transcriptase (YSB_LS_CL1205Contig1). The insect adipokinetic hormones known to be associated with energy-requiring activities like flight and locomotion in *D. melanogaster* and *B. mori* (Staubli *et al.* 2002) might be operating in YSB also. The YSB-specific CarE and CYP450 might be involved in key mechanisms of resistance to insecticides (particularly OPs and pyrethroids), as reported in *S. litura* (Wang *et al.* 2015). It can be postulated that the specific ZF protein and leucine-zipper TFs might play role in gene regulation. The presence of unique transcripts encoding titin-like protein, muscle M-line assembly protein, U5 small nuclear ribonucleoprotein, and mitochondrial carrier protein suggests that they have a crucial role in basic regulatory activities like RNA splicing, flight, and insect muscle formation.

### Conclusions

Comprehensive transcriptome data of four larval developmental stages of YSB was generated, which greatly enriches the currently available genomic resource for this organism. Differentially expressed transcripts revealed the phenology and behavioral characteristics of different larval stages of YSB. Although there are many transcripts that are present in all the stages, stage-specific transcripts might have a greater role in specific larval development. It appears that YSB has evolved independently from other lepidopteron pests by having specific mechanisms for visual recognition as well as chemoreception, which might play a role in host recognition and have a molecular basis for host specificity. The role of hormones and their regulation in moulting and development was clearly observed. The novel transcripts of YSB identified in the present study could be efficiently used as candidate genes for knocking-down or editing to use in pest management strategies. The presence of *Dcr-1*, *Dcr-2*, *Ago-1*, *Ago-2*, *Sid-1*, and *Sid1*-related genes provided strong evidence of RNAi machinery in YSB. Further, YSB EST-SSR markers are new additions to the genomic resources of YSB that can be readily used for genetic diversity studies. Overall, the data provides information on the larval development of an agriculturally important pest. The wealth of data generated on the genes involved in important molecular mechanisms will facilitate further research into the developmental biology of YSB as well as the evolution of novel pest management strategies.

## Supplementary Material

Supplemental material is available online at www.g3journal.org/lookup/suppl/doi:10.1534/g3.117.043737/-/DC1.

## References

[bib1] Andersen S. O. Hojrup P. Roepstorff P., 1995 Insect cuticular proteins. Insect Biochem. Mol. Biol. 25: 153–176. 7711748 10.1016/0965-1748(94)00052-j

[bib2] Arias R. S. Blanco C. A. Portilla M. Snodgrass G. L. Scheffler B. E., 2011 First microsatellites from *Spodoptera frugiperda* (Lepidoptera: Noctuidae) and their potential use for population genetics. Ann. Entomol. Soc. Am. 104: 576–587.

[bib3] Aronstein K. Pankiw T. Saldivar E., 2006 SID-1 is implicated in systemic gene silencing in the honey bee. J. Apic. Res. 45: 20–24.

[bib4] Banerjee S. N. Pramanik L. M., 1967 The lepidopterous stalk borers of rice and their life cycles in the tropics, pp. 103–124 in *The Major Insect Pests of the Rice Plant*. The Johns Hopkins Press, Baltimore, MD.

[bib5] Bauer M. Katzenberger J. D. Hamm A. C. Bonaus M. Zinke I., 2006 Purine and folate metabolism as a potential target of sex-specific nutrient allocation in *Drosophila* and its implication for lifespan-reproduction tradeoff. Physiol. Genomics 25: 393–404. 16569777 10.1152/physiolgenomics.00009.2006

[bib6] Beermann A. Aranda M. Schroder R., 2004 The Sp8 zinc-finger transcription factor is involved in allometric growth of the limbs in the beetle *Tribolium castaneum*. Development 131: 733–742. 14724124 10.1242/dev.00974

[bib7] Belles X. Martin D. Piulachs M. D., 2005 The mevalonate pathway and the synthesis of juvenile hormone in insects. Annu. Rev. Entomol. 50: 181–199. 15355237 10.1146/annurev.ento.50.071803.130356

[bib8] Bengtsson J. M. Trona F. Montagne N. Anfora G. Ignell R., 2012 Putative chemosensory receptors of the codling moth, *Cydia pomonella*, identified by antennal transcriptome analysis. PLoS One 7: e31620. 22363688 10.1371/journal.pone.0031620 PMC3282773

[bib9] Camargo R. D. A. Herai R. H. Santos L. N. Bento F. M. Lima J. E., 2015 *De novo* transcriptome assembly and analysis to identify potential gene targets for RNAi-mediated control of the tomato leafminer (*Tuta absoluta*). BMC Genomics 16: 635. 26306628 10.1186/s12864-015-1841-5 PMC4550053

[bib10] Chelliah A. Benthur J. S. Prakasa Rao P. S., 1989 Approaches to rice management-achievements and opportunities. Oryzae 26: 12–26.

[bib11] Chippendale G. M., 1977 Hormonal regulation of larval diapauses. Annu. Rev. Entomol. 22: 121–138.

[bib12] Conesa A. Gotz S. Garcia-Gomez J. M. Teroll J. Talon M., 2005 Blast2GO: a universal tool for annotation, visualization and analysis in functional genomics research. Bioinformatics 21: 3674–3676. 16081474 10.1093/bioinformatics/bti610

[bib13] Cruz J. Mane-Padros D. Belles X. Martin D., 2006 Functions of the ecdysone receptor isoform-A in the hemimetabolous insect *Blattella germanica* revealed by systemic RNAi *in vivo*. Dev. Biol. 297: 158–171. 16890931 10.1016/j.ydbio.2006.06.048

[bib14] Cui F. Lin Z. Wang H. Liu S. Chang H., 2011 Two single mutations commonly cause qualitative change of nonspecific carboxylesterases in insects. Insect Biochem. Mol. Biol. 41: 1–8. 20888910 10.1016/j.ibmb.2010.09.004

[bib15] David J. P. Strode C. Vontas J. Nikou D. Vaughan A., 2005 The *Anopheles gambiae* detoxification chip: a highly specific microarray to study metabolic-based insecticide resistance in malaria vectors. Proc. Natl. Acad. Sci. USA 102: 4080–4084. 15753317 10.1073/pnas.0409348102 PMC554807

[bib16] Enayati A. A. Ranson H. Hemingway J., 2005 Insect glutathione transferases and insecticide resistance. Insect Mol. Biol. 14: 3–8. 15663770 10.1111/j.1365-2583.2004.00529.x

[bib17] Feuda R. Marletaz F. Bentley M. A. Holland P. W., 2016 Conservation, duplication, and divergence of five opsin genes in insect evolution. Genome Biol. Evol. 8: 579–587. 26865071 10.1093/gbe/evw015 PMC4824169

[bib18] Gong D. P. Zhang H. J. Zhao P. Xia Q. Y. Xiang Z. H., 2009 The Odorant binding protein gene family from the genome of silkworm, *Bombyx mori*. BMC Genomics 10: 332. 19624863 10.1186/1471-2164-10-332 PMC2722677

[bib19] Gong L. Wang Z. Wang H. Qi J. Hu M., 2015 Core RNAi machinery and three Sid-1 related genes in *Spodoptera litura* (Fabricius). Int. J. Agric. Biol. 17: 937–944.

[bib20] Grabherr M. G. Haas B. J. Yassour M. Levin J. Z. Thompson D. A., 2011 Trinity: reconstructing a full-length transcriptome without a genome from RNA-Seq data. Nat. Biotechnol. 29: 644–652. 21572440 10.1038/nbt.1883 PMC3571712

[bib21] Grosse-Wilde E. Kuebler L. S. Bucks S. Vogel H. Wicher D., 2011 Antennal transcriptome of *Manduca sexta*. Proc. Natl. Acad. Sci. USA 108: 7449–7454. 21498690 10.1073/pnas.1017963108 PMC3088587

[bib22] Haas B. J. Zody M. C., 2010 Advancing RNA-Seq analysis. Nat. Biotechnol. 28: 421–423. 20458303 10.1038/nbt0510-421

[bib23] Haas B. J. Papanicolaou A. Yassour M. Grabherr M. Blood P. D., 2013 *De novo* transcript sequence reconstruction from RNA-Seq: reference generation and analysis with Trinity. Nat. Protoc. 8: 1494–1512. 23845962 10.1038/nprot.2013.084 PMC3875132

[bib24] Hamarsheh O. Amro A., 2011 Characterization of simple sequence repeats (SSRs) from *Phlebotomus papatasi* (Diptera: Psychodidae) expressed sequence tags (ESTs). Parasit. Vectors 4: 189. 21958493 10.1186/1756-3305-4-189 PMC3191335

[bib25] Hammond S. M. Boettcher S. Caudy A. A. Kobayashi R. Hannon G. J., 2001 Argonaute2, a link between genetic and biochemical analyses of RNAi. Science 293: 1146–1150. 11498593 10.1126/science.1064023

[bib26] He W. You M. Vasseur L. Yang G. Xie M., 2012 Developmental and insecticide-resistant insights from the *de novo* assembled transcriptome of the diamondback moth, *Plutella xylostella*. Genomics 99: 169–177. 22240003 10.1016/j.ygeno.2011.12.009

[bib27] Heidari R. Devonshire A. L. Campbell B. E. Dorrian S. J. Oakeshott J. G., 2005 Hydrolysis of pyrethroids by carboxylesterases from *Lucilia cuprina* and *Drosophila melanogaster* with active sites modified by in vitro mutagenesis. Insect Biochem. Mol. Biol. 35: 597–609. 15857765 10.1016/j.ibmb.2005.02.018

[bib28] Huvenne H. Smagghe G., 2012 Mechanisms of dsRNA uptake in insects and potential of RNAi for pest control: a review. J. Insect Physiol. 56: 227–235. 10.1016/j.jinsphys.2009.10.004 19837076

[bib29] Jia X. J. Wang H. X. Yan Z. G. Zhang M. Z. Wei C. H., 2016 Antennal transcriptome and differential expression of olfactory genes in the yellow peach moth, *Conogethes punctiferalis* (Lepidoptera: Crambidae). Sci. Rep. 6: 29067. 27364081 10.1038/srep29067 PMC4929561

[bib30] Jones A. K. Raymond-Delpech V. Thany S. H. Gauthier M. Sattelle D. B., 2006 The nicotinic acetylcholine receptor gene family of the honey bee, *Apis mellifera*. Genome Res. 16: 1422–1430. 17065616 10.1101/gr.4549206 PMC1626644

[bib31] Kageyama R. Nakanishi S., 1997 Helix-loop-helix factors in growth and differentiation of the vertebrate nervous system. Curr. Opin. Genet. Dev. 7: 659–665. 9388783 10.1016/s0959-437x(97)80014-7

[bib32] Kalia R. K. Rai M. K. Kalia S. Singh R. Dhawan A. K., 2011 Microsatellite markers: an overview of the recent progress in plants. Euphytica 177: 309–334.

[bib33] Kobayashi I. Tsukioka H. Komoto N. Uchino K. Sezutsu H., 2012 SID-1 protein of *Caenorhabditis elegans* mediates uptake of dsRNA into *Bombyx* cells. Insect Biochem. Mol. Biol. 42: 148–154. 22178129 10.1016/j.ibmb.2011.11.007

[bib34] Kola V. S. Renuka P. Madhav M. S. Mangrauthia S. K., 2015 Key enzymes and proteins of crop insects as candidate for RNAi based gene silencing. Front. Physiol. 6: 119. 25954206 10.3389/fphys.2015.00119 PMC4406143

[bib35] Kola V. S. R. Renuka P. Padmakumari A. P. Mangrauthia S. K. Balachandran S. M., 2016 Silencing of CYP6 and APN genes affects the growth and development of rice yellow stem borer, *Scirpophaga incertulas*. Front. Physiol. 7: 20. 26903874 10.3389/fphys.2016.00020 PMC4751738

[bib36] Kulkarni K. S. Zala H. N. Bosamia T. C. Shukla Y. M. Joshi C. G., 2016 *De novo* transcriptome sequencing to dissect candidate genes associated with pearl millet-downy mildew (*Sclerospora graminicola Sacc*.) interaction. Front. Plant Sci. 7: 847. 27446100 10.3389/fpls.2016.00847 PMC4916200

[bib37] Laity J. H. Lee B. M. Wright P. E., 2001 Zinc finger proteins: new insights into structural and functional diversity. Curr. Opin. Struct. Biol. 11: 39–46. 11179890 10.1016/s0959-440x(00)00167-6

[bib38] Leal W. S., 2013 Odorant reception in insects: roles of receptors, binding proteins, and degrading enzymes. Annu. Rev. Entomol. 58: 373–391. 23020622 10.1146/annurev-ento-120811-153635

[bib39] Li B. Predel R. Neupert S. Hauser F. Tanaka Y., 2008 Genomics, transcriptomics and peptidomics of neuropeptides and protein hormones in the red flour beetle *Tribolium castaneum*. Genome Res. 18: 113–122. 18025266 10.1101/gr.6714008 PMC2134770

[bib40] Li L. T. Zhu Y. B. Ma J. F. Li Z. Y. Dong Z. P., 2013 An analysis of the *Athetis lepigone* transcriptome from four developmental stages. PLoS One 8: e73911. 24058501 10.1371/journal.pone.0073911 PMC3772797

[bib41] Li S. W. Yang H. Liu Y. F. Liao Q. R. Du J., 2012 Transcriptome and gene expression analysis of the rice leaf folder, *Cnaphalocrosis medinalis*. PLoS One 7: e47401. 23185238 10.1371/journal.pone.0047401 PMC3501527

[bib42] Li W. Godzik A., 2006 Cd-hit: a fast program for clustering and comparing large sets of protein or nucleotide sequences. Bioinformatics 22: 1658–1659. 16731699 10.1093/bioinformatics/btl158

[bib43] Li X. Schuler M. A. Berenbaum M. R., 2007 Molecular mechanisms of metabolic resistance to synthetic and natural xenobiotics. Annu. Rev. Entomol. 52: 231–253. 16925478 10.1146/annurev.ento.51.110104.151104

[bib44] Listinger J. A., 1979 Major insect–pests of rainfed wetland rice, Tropical Asia. Int. Rice Res. Newslett. 4: 14–15.

[bib45] Liu Y. Gu S. Zhang Y. Guo Y. Wang G., 2012 Candidate olfaction genes identified within the *Helicoverpa armigera* antennal transcriptome. PLoS One 7: e48260. 23110222 10.1371/journal.pone.0048260 PMC3482190

[bib46] Ma W. Zhang Z. Peng C. Wang X. Li F., 2012 Exploring the midgut transcriptome and brush border membrane vesicle proteome of the rice stem borer, *Chilo suppressalis* (Walker). PLoS One 7: e38151. 22666467 10.1371/journal.pone.0038151 PMC3362559

[bib47] McBrayer Z. Ono H. Shimell M. Parvy J. P. Beckstead R. B., 2007 Prothoracicotropic hormone regulates developmental timing and body size in *Drosophila*. Dev. Cell 13: 857–871. 18061567 10.1016/j.devcel.2007.11.003 PMC2359579

[bib48] Metzgar D. Bytof J. Wills C., 2000 Selection against frame shift mutations limits microsatellite expansion in coding DNA. Genome Res. 10: 72–80. 10645952 PMC310501

[bib49] Moore A. W. Barbel S. Jan L. Y. Jan Y. N., 2000 A genome wide survey of basic helix–loop–helix factors in *Drosophila*. Proc. Natl. Acad. Sci. USA 97: 10436–10441. 10973473 10.1073/pnas.170301897 PMC27042

[bib50] Muralidharan K. Pasalu I. C., 2006 Assessments of crop losses in rice ecosystems due to stem borer damage (Lepidoptera: Pyralidae). Crop Prot. 25: 409–417.

[bib51] Ningshen T. J. Aparoy P. Ventaku V. R. Dutta-Gupta A., 2013 Functional interpretation of a non-gut hemocoelic tissue aminopeptidase N (APN) in a lepidopteran insect pest *Achaea janata*. PLoS One 8: e79468. 24244508 10.1371/journal.pone.0079468 PMC3828369

[bib52] Ortelli F. Rossiter L. C. Vontas J. Ranson H. Hemingway J., 2003 Heterologous expression of four glutathione transferase genes genetically linked to a major insecticide-resistance locus from the malaria vector *Anopheles gambiae*. Biochem. J. 373: 957–963. 12718742 10.1042/BJ20030169 PMC1223529

[bib53] Ota A. Kusakabe T. Sugimoto Y. Takahashi M. Nakajima Y., 2002 Cloning and characterization of testis-specific tektin in *Bombyx mori*. Comp. Biochem. Physiol. B Biochem. Mol. Biol. 133: 371–382. 12431405 10.1016/s1096-4959(02)00153-7

[bib54] Ou J. Deng H. M. Zheng S. C. Huang L. H. Feng Q., 2014 Transcriptomic analysis of developmental features of *Bombyx mori* wing disc during metamorphosis. BMC Genomics 15: 1–35. 25261999 10.1186/1471-2164-15-820 PMC4196006

[bib55] Ozsolak F. Milos P. M., 2011 RNA sequencing: advances, challenges and opportunities. Nat. Rev. Genet. 12: 87–98. 21191423 10.1038/nrg2934 PMC3031867

[bib56] Padmakumari A. P. Katti G. Sailaja V. Padmavathi Ch. Lakshmi V. J., 2013 Delineation of larval instars in field populations of rice yellow stem borer, *Scirpophaga incertulas* (Walk.). Oryza 50: 259–267.

[bib57] Parekh M. J. Kumar S. Zala H. N. Fougat R. S. Patel C. B., 2016 Development and validation of novel fiber relevant dbEST–SSR markers and their utility in revealing genetic diversity in diploid cotton (*Gossypium herbaceum* and *G. arboreum*). Ind. Crops Prod. 83: 620–629.

[bib58] Park Y. Kim Y. J. Dupriez V. Adams M. E., 2003 Two subtypes of ecdysis-triggering hormone receptor in *Drosophila melanogaster*. J. Biol. Chem. 278: 17710–17715. 12586820 10.1074/jbc.M301119200

[bib59] Patel R. K. Jain M., 2012 NGS QC toolkit: a toolkit for quality control of next generation sequencing data. PLoS One 7: e30619. 22312429 10.1371/journal.pone.0030619 PMC3270013

[bib60] Pathak M. D. Khan Z. R., 1994 Insect Pests of Rice. International Rice Research Institute, Manila, Philippines.

[bib61] Patnaik B. B. Park S. Y. Kang S. W. Wang H. J. H. Wang T. H., 2016 Transcriptome profile of the Asian giant hornet (*Vespa mandarinia*) using Illumina HiSeq 4000 sequencing: *de novo* assembly, functional annotation and discovery of SSR markers. Int. J. Genomics 2016: 4169587. 26881195 10.1155/2016/4169587 PMC4736913

[bib62] Pertea G. Huang X. Liang F. Antonescu V. Sultana R., 2003 TIGR gene indices clustering tools (TGICL): a software system for fast clustering of large EST datasets. Bioinformatics 19: 651–652. 12651724 10.1093/bioinformatics/btg034

[bib63] Porreca G. J. Zhang K. Li J. B. Xie B. Austin D., 2007 Multiplex amplification of large sets of human exons. Nat. Methods 4: 931–936. 17934468 10.1038/nmeth1110

[bib64] Posnien N. Schinko J. Grossmann D. Shippy T. D. Konopova B., 2009 RNAi in the red flour beetle (*Tribolium*). Cold Spring Harb. Protoc. 8: 5256. 10.1101/pdb.prot5256 20147232

[bib65] Prentice K. Pertry I. Christiaens O. Bauters L. Bailey A., 2015 Transcriptome analysis and systemic RNAi response in the African sweetpotato Weevil (*Cylas puncticollis*, Coleoptera, Brentidae). PLoS One 10: e0115336. 25590333 10.1371/journal.pone.0115336 PMC4295849

[bib66] Pridgeon J. W. Zhang L. Liu N., 2003 Over expression of CYP4G19 associated with a pyrethroid-resistant strain of the German cockroach, *Blattella germanica* (L.). Gene 314: 157–163. 14527728 10.1016/s0378-1119(03)00725-x

[bib67] Ranson H. Claudianos C. Ortelli F. Abgrall C. Hemingway J., 2002 Evolution of supergene families associated with insecticide resistance. Science 298: 179–181. 12364796 10.1126/science.1076781

[bib68] Rath P. C., 2001 Efficacy of insecticides, neem and Bt formulation against stem borer on rice yield in West Bengal. J. Appl. Zool. Res. 12: 191–193.

[bib69] Raymond-Delpech V. Matsuda K. Sattelle B. M. Rauh J. J. Sattelle D. B., 2005 Ion channels: molecular targets of neuroactive insecticides. Invert. Neurosci. 5: 119–133. 16172884 10.1007/s10158-005-0004-9

[bib70] Robinson M. D. McCarthy D. J. Smyth G. K., 2010 edgeR: a bioconductor package for differential expression analysis of digital gene expression data. Bioinformatics 26: 139–140. 19910308 10.1093/bioinformatics/btp616 PMC2796818

[bib71] Rogers B. T. Peterson M. D. Kaufman T. C., 2002 The development and evolution of insect mouthparts as revealed by the expression patterns of gnathocephalic genes. Evol. Dev. 4: 96–110. 12004967 10.1046/j.1525-142x.2002.01065.x

[bib72] Saeed A. I. Sharov V. White J. Li J. Liang W., 2003 TM4: a free, open-source system for microarray data management and analysis. Biotechniques 34: 374–378. 12613259 10.2144/03342mt01

[bib73] Sato K. Pellegrino M. Nakagawa T. Nakagawa T. Vosshall L. B., 2008 Insect olfactory receptors are heteromeric ligand-gated ion channels. Nature 452: 1002–1006. 18408712 10.1038/nature06850

[bib74] Schliesky S. Gowik U. Weber A. P. M. Brautigam A., 2012 RNA-seq assembly – are we there yet? Front. Plant Sci. 3: 220. 23056003 10.3389/fpls.2012.00220 PMC3457010

[bib75] Schmittgen T. D. Livak K. J., 2008 Analyzing real-time PCR data by the comparative CT method. Nat. Protoc. 3: 1101–1108. 18546601 10.1038/nprot.2008.73

[bib76] Scott J. G., 2008 Insect cytochrome P450s: thinking beyond detoxification, Vol. 1, pp. 117–124 in *Recent Advances in Insect Physiology*, *Toxicology and Molecular Biology*, edited by Liu N. Research Signpost, Kerala, India.

[bib77] Shao Y. M. Dong K. Zhang C. X., 2007 The nicotinic acetylcholine receptor gene family of the silkworm, *Bombyx mori*. BMC Genomics 8: 1. 17868469 10.1186/1471-2164-8-324 PMC2045683

[bib78] Smith B. H. Getz W. M., 1994 Nonpheromonal olfactory processing in insects. Annu. Rev. Entomol. 39: 351–375.

[bib79] Staubli F. Jorgensen T. J. Cazzamali G. Williamson M. Lenz C., 2002 Molecular identification of the insect adipokinetic hormone receptors. Proc. Natl. Acad. Sci. USA 99: 3446–3451. 11904407 10.1073/pnas.052556499 PMC122543

[bib80] Stevens J. L. Snyder M. J. Koener J. F. Feyereisen R., 2000 Inducible P450s of the CYP9 family from larval *Manduca sexta* midgut. Insect Biochem. Mol. Biol. 30: 559–568. 10844248 10.1016/s0965-1748(00)00024-2

[bib81] Stolle E. Kidner J. H. Moritz R. F., 2013 Patterns of evolutionary conservation of microsatellites (SSRs) suggest a faster rate of genome evolution in Hymenoptera than in Diptera. Genome Biol. Evol. 5: 151–162. 23292136 10.1093/gbe/evs133 PMC3595035

[bib82] Tamura K. Dudley J. Nei M. Kumar S., 2007 MEGA4: molecular evolutionary genetics analysis (MEGA) software version 4.0. Mol. Biol. Evol. 24: 1596–1599. 17488738 10.1093/molbev/msm092

[bib83] Tanaka Y. Suetsugu Y. Yamamoto K. Noda H. Shinoda T., 2014 Transcriptome analysis of neuropeptides and G-protein coupled receptors (GPCRs) for neuropeptides in the brown planthopper *Nilaparvata lugens*. Peptides 53: 125–133. 23932938 10.1016/j.peptides.2013.07.027

[bib84] Tomoyasu Y. Miller S. C. Tomita S. Schoppmeier M. Grossmann D., 2008 Exploring systemic RNA interference in insects: a genome-wide survey for RNAi genes in *Tribolium*. Genome Biol. 9: R10. 18201385 10.1186/gb-2008-9-1-r10 PMC2395250

[bib85] Vogel H. Badapanda C. Knorr E. Vilcinskas A., 2014 RNA-sequencing analysis reveals abundant developmental stage-specific and immunity-related genes in the pollen beetle *Meligethes aeneus*. Insect Mol. Biol. 23: 98–112. 24252113 10.1111/imb.12067

[bib86] Vontas J. G. Small G. J. Hemingway J., 2001 Glutathione S-transferases as antioxidant defense agents confer pyrethroid resistance in *Nilaparvata lugens*. Biochem. J. 357: 65–72. 11415437 10.1042/0264-6021:3570065 PMC1221929

[bib87] Wang P. Zhang X. Zhang J., 2005 Molecular characterization of four midgut aminopeptidase N isozymes from the cabbage looper, *Trichoplusia ni*. Insect Biochem. Mol. Biol. 35: 611–620. 15857766 10.1016/j.ibmb.2005.02.002

[bib88] Wang R. L. Staehelin C. Xia Q.-Q. Su Y.-J. Zeng R.-S., 2015 Identification and characterization of CYP9A40 from the tobacco cutworm moth (*Spodoptera litura*), a Cytochrome P450 gene induced by plant allelochemicals and insecticides. Int. J. Mol. Sci. 16: 22606–22620. 26393579 10.3390/ijms160922606 PMC4613326

[bib89] Wang Y. Zhang H. Li H. Miao X., 2011 Second-generation sequencing supply an effective way to screen RNAi targets in large scale for potential application in pest insect control. PLoS One 6: e18644. 21494551 10.1371/journal.pone.0018644 PMC3073972

[bib90] Wolf J. B. W., 2013 Principles of transcriptome analysis and gene expression quantification, an RNA-seq tutorial. Mol. Ecol. Resour. 13: 559–572. 23621713 10.1111/1755-0998.12109

[bib91] Wolfe S. A. Nekludova L. Pabo C. O., 2000 DNA recognition by Cys2His2 zinc finger proteins. Annu. Rev. Biophys. Biomol. Struct. 29: 183–212. 10940247 10.1146/annurev.biophys.29.1.183

[bib92] Xu H. J. Chen T. Ma X. F. Xue J. Pan P. L., 2013 Genome‐wide screening for components of small interfering RNA (siRNA) and micro‐RNA (miRNA) pathways in the brown plant hopper, *Nilaparvata lugens* (Hemiptera: Delphacidae). Insect Mol. Biol. 22: 635–647. 23937246 10.1111/imb.12051

[bib93] Xue J. Zhou X. Zhang C. X. Yu L. L. Fan H. W., 2014 Genomes of the rice pest brown plant hopper and its endosymbionts reveal complex complementary contributions for host adaptation. Genome Biol. 15: 521. 25609551 10.1186/s13059-014-0521-0 PMC4269174

[bib94] Yamamoto K. Shigeoka Y. Aso Y. Banno Y. Kimura M., 2009 Molecular and biochemical characterization of a Zeta-class glutathione S-transferase of the silk moth. Pestic. Biochem. Physiol. 94: 30–35.

[bib95] Yan S. Cui F. Qiao C., 2009 Structure, function and applications of carboxylesterases from insects for insecticide resistance. Protein Pept. Lett. 6: 1181–1188. 10.2174/092986609789071243 19508184

[bib96] Yan S. Zhu J. Zhu W. Zhang X. Li Z., 2014 The expression of three opsin genes from the compound eye of *Helicoverpa armigera* (Lepidoptera: Noctuidae) is regulated by a circadian clock, light conditions and nutritional status. PLoS One 9: e111683. 25353953 10.1371/journal.pone.0111683 PMC4213014

[bib97] Yang X. M. Sun J.-T. Xue X. F. Zhu W. C. Hong X. Y., 2012 Development and characterization of 18 novel EST-SSRs from the Western flower Thrips, *Frankliniella occidentalis* (Pergande). Int. J. Mol. Sci. 13: 2863–2876. 22489130 10.3390/ijms13032863 PMC3317692

[bib98] Yin C. Liu Y. Liu J. Xiao H. Huang S., 2014 ChiloDB: a genomic and transcriptome database for an important rice insect pest *Chilo suppressalis*. Database 2014: bau065. 24997141 10.1093/database/bau065 PMC4082153

[bib99] You F. M. Huo N. Gu Y. Q. Luo M. C. Ma Y., 2008 BatchPrimer3: a high throughput web application for PCR and sequencing primer design. BMC Bioinformatics 9: 253. 18510760 10.1186/1471-2105-9-253 PMC2438325

[bib100] Yu R. Xu X. Liang Y. Tian H. Pan Z., 2014 The insect ecdysone receptor is a good potential target for RNAi-based pest control. Int. J. Biol. Sci. 10: 1171–1180. 25516715 10.7150/ijbs.9598 PMC4261201

[bib101] Zhang Y. Zheng Y. Li D. Fan Y., 2014 Transcriptomics and identification of the chemoreceptor superfamily of the pupal parasitoid of the oriental fruit fly, *Spalangia endius* Walker (Hymenoptera: Pteromalidae). PLoS One 9: e87800. 24505315 10.1371/journal.pone.0087800 PMC3914838

[bib102] Zhao W. Lu L. Yang P. Cui N. Kang L., 2016 Organ-specific transcriptome response of the small brown plant hopper toward rice stripe virus. Insect Biochem. Mol. Biol. 70: 60–72. 26678499 10.1016/j.ibmb.2015.11.009

[bib103] Zhao X. M. Liu C. Li Q. Y. Hu W. B. Zhou M. T., 2014 Basic helix-loop-helix transcription factor Bmsage is involved in regulation of fibroin H-chain gene via interaction with SGF1 in *Bombyx mori*. PLoS One 9: e94091. 24740008 10.1371/journal.pone.0094091 PMC3989216

[bib104] Zhou X. J. Sheng C. F. Li M. Wan H. Liu D., 2010 Expression responses of nine cytochrome P450 genes to xenobiotics in the cotton bollworm *Helicoverpa armigera*. Pestic. Biochem. Physiol. 97: 209–213.

[bib105] Zhu J. Q. Liu S. Ma Y. Zhang J. Q. Qi H. S., 2012 Improvement of pest resistance in transgenic tobacco plants expressing dsRNA of an insect-associated gene EcR. PLoS One 7: e38572. 22685585 10.1371/journal.pone.0038572 PMC3369839

