# Supplementary figures and images for: RNA-seq of Rice Yellow Stem Borer Scirpophaga incertulas Reveals Molecular Insights During Four Larval Developmental Stages
Source: G3 (Bethesda). 2017 Jul 17;7(9):3031–45. doi: 10.1534/g3.117.043737 (PMC5592929; doi:10.1534/g3.117.043737)

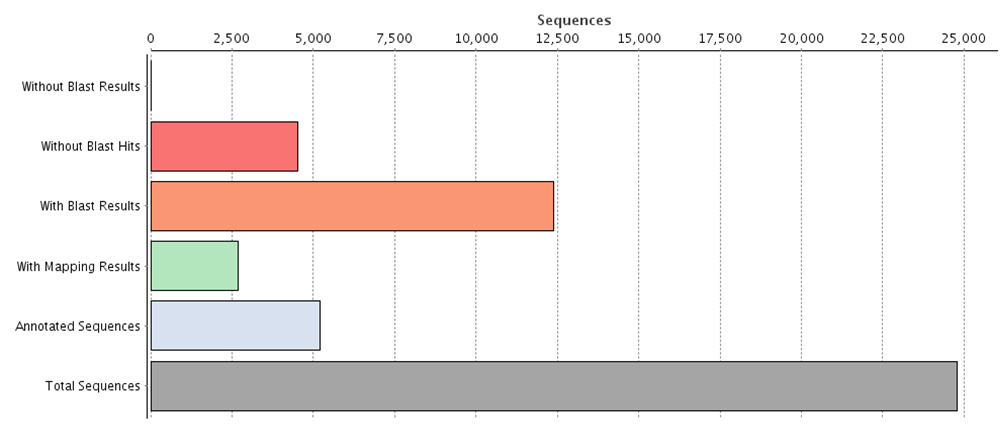

Supplement: Supplementary file 1 [file 3031FigureS1.tif]

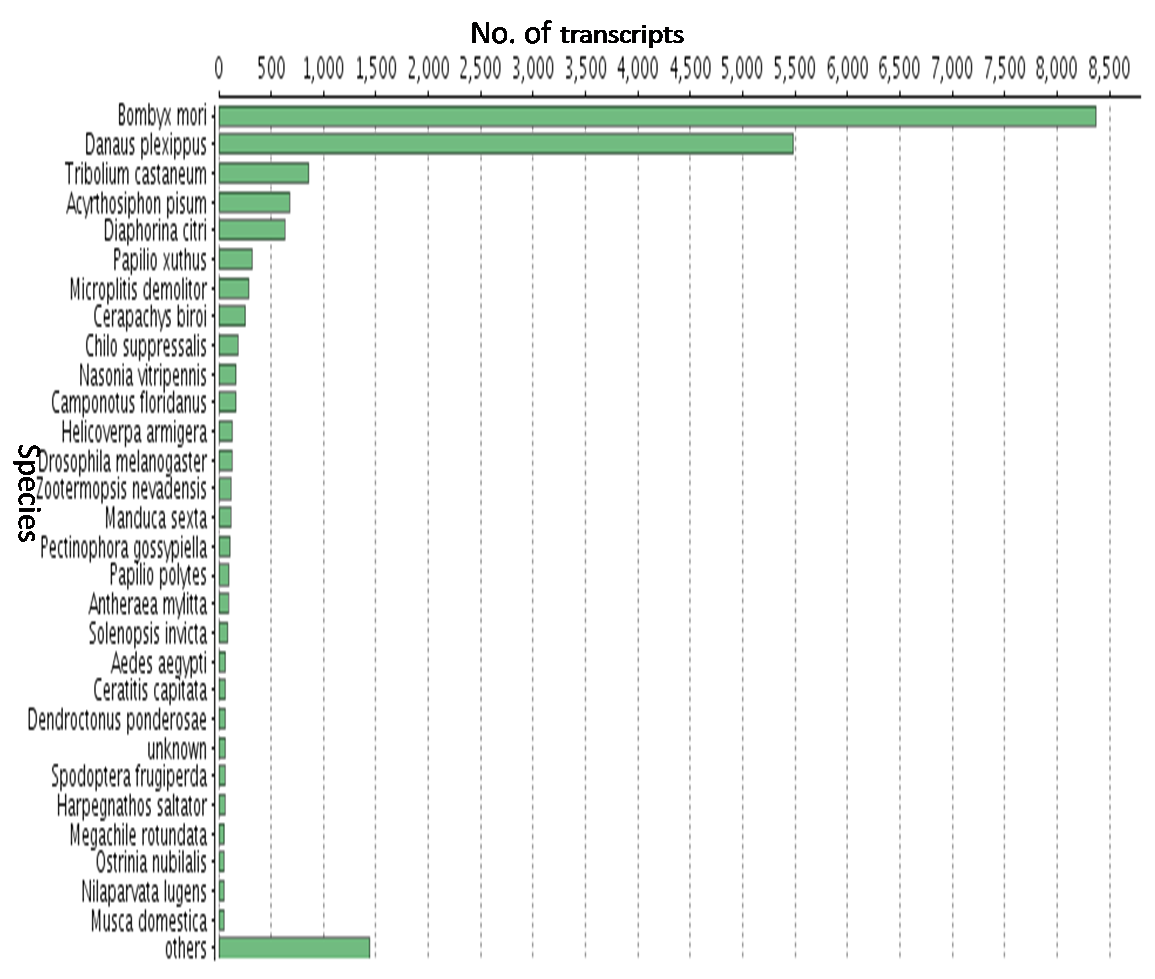

Supplement: Supplementary file 2 [file 3031FigureS2.tif]

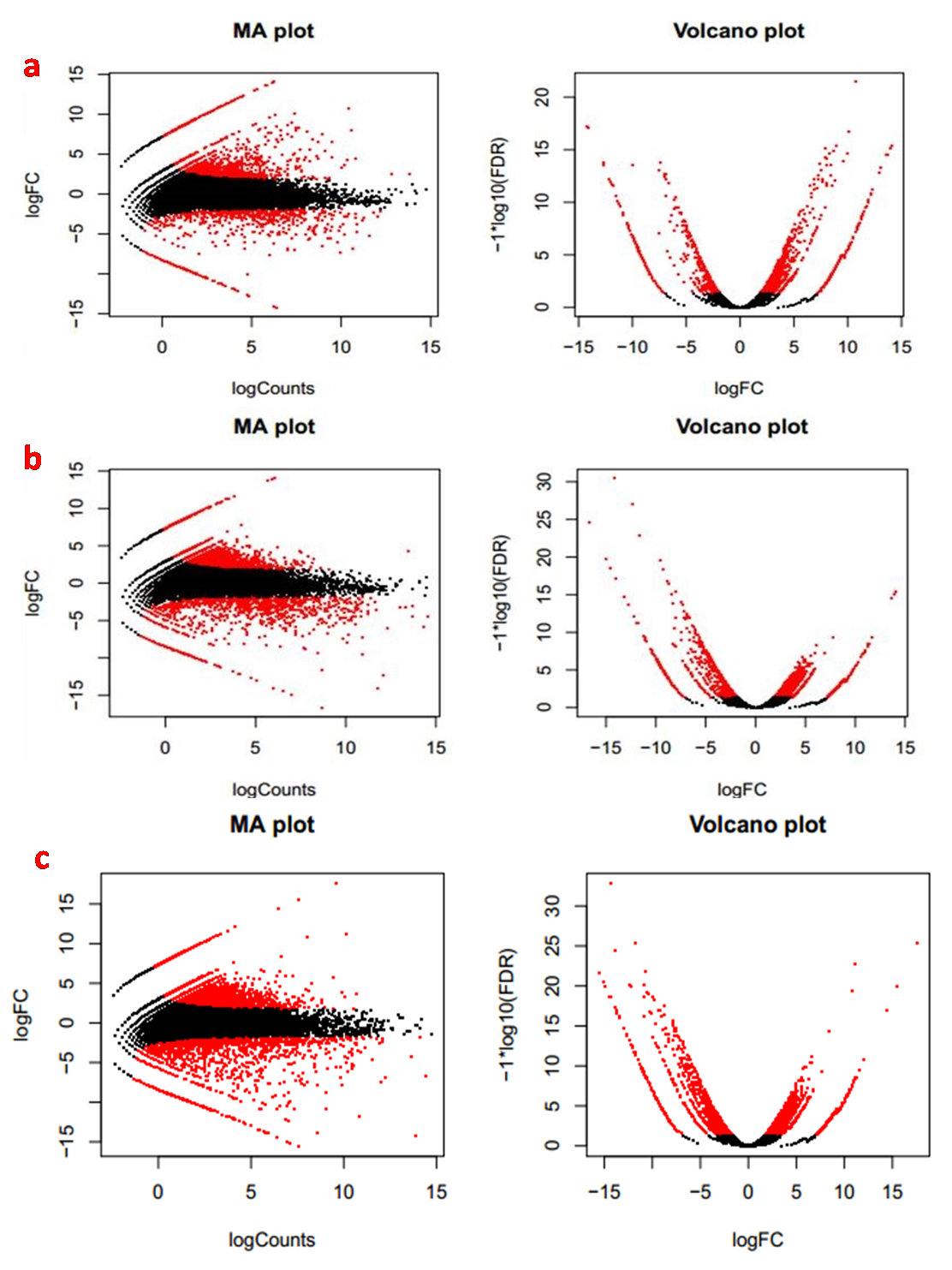

Supplement: Supplementary file 3 [file 3031FigureS3.tif]
